# Supplementary material for: Transcriptomic analysis of tissue-resident memory T cells of the fallopian tube reveals a precursor immune surveillance network for ovarian cancer prevention
Source: Nat Commun. 2026 Jul 13;17:8617. doi: 10.1038/s41467-026-74599-4 (PMC13486501; doi:10.1038/s41467-026-74599-4)
Supplement: Supplementary file 1 [file 41467_2026_74599_MOESM1_ESM.pdf]

Supplementary Information

Transcriptomic analysis of tissue-resident memory T cells of the fallopian tube reveals a precursor immune surveillance network for ovarian cancer prevention.

Luyao Wang[1,2], Breeshey Roskams-Hieter[2,3,4], Nosheen Hussain[1,2,4], Joel Nulsen[4], Aneesh Aggarwal[1,2], May Sallam[1,2,5], Lili Wang[4], Lena Rai[1,2], Hui Shi[1,2], Amro Ahmed-Ebbiary[4], Aws Al-deka[4,6], Takashi Takeda[1,2], Mara Artibani[1,2], Hooman Soleymani majd[7], Jason Yap[8], Christopher Yau[2], Nancy Zaarour[1,2*], Ahmed Ashour Ahmed[1,2**]

[1]Ovarian Cancer Cell Laboratory, MRC Weatherall Institute of Molecular Medicine, University of Oxford, Oxford OX3 9DS, UK
[2]Nuffield Department of Women's & Reproductive Health, University of Oxford, Oxford OX3 9DU, UK
[3]EMBL-EBI, Wellcome Genome Campus, Hinxton, Cambridgeshire, CB10 1SD, UK
[4]Singula Bio, Oxford, OX1 3LE
[5]Human Anatomy and Embryology Department, Faculty of Medicine, Mansoura University, Mansoura, Egypt
[6]Department of Pathology, University of Cambridge, Cambridge, United Kingdom
[7]Department of Gynecological Oncology, Churchill Hospital, Oxford University Hospitals, Oxford OX3 7LE, UK
[8]Pan Birmingham Gynaecological Cancer Centre, Midland Metropolitan University Hospital, Grove Lane, Smethwick B66 2QT, United Kingdom
*Correspondence: nancy.zaarour@lincoln.ox.ac.uk
**Correspondence: ahmed.ahmed@wrh.ox.ac.uk

This PDF file includes:

Supplementary Figures 1 to 9

Supplementary Table 1

Supplementary Figures

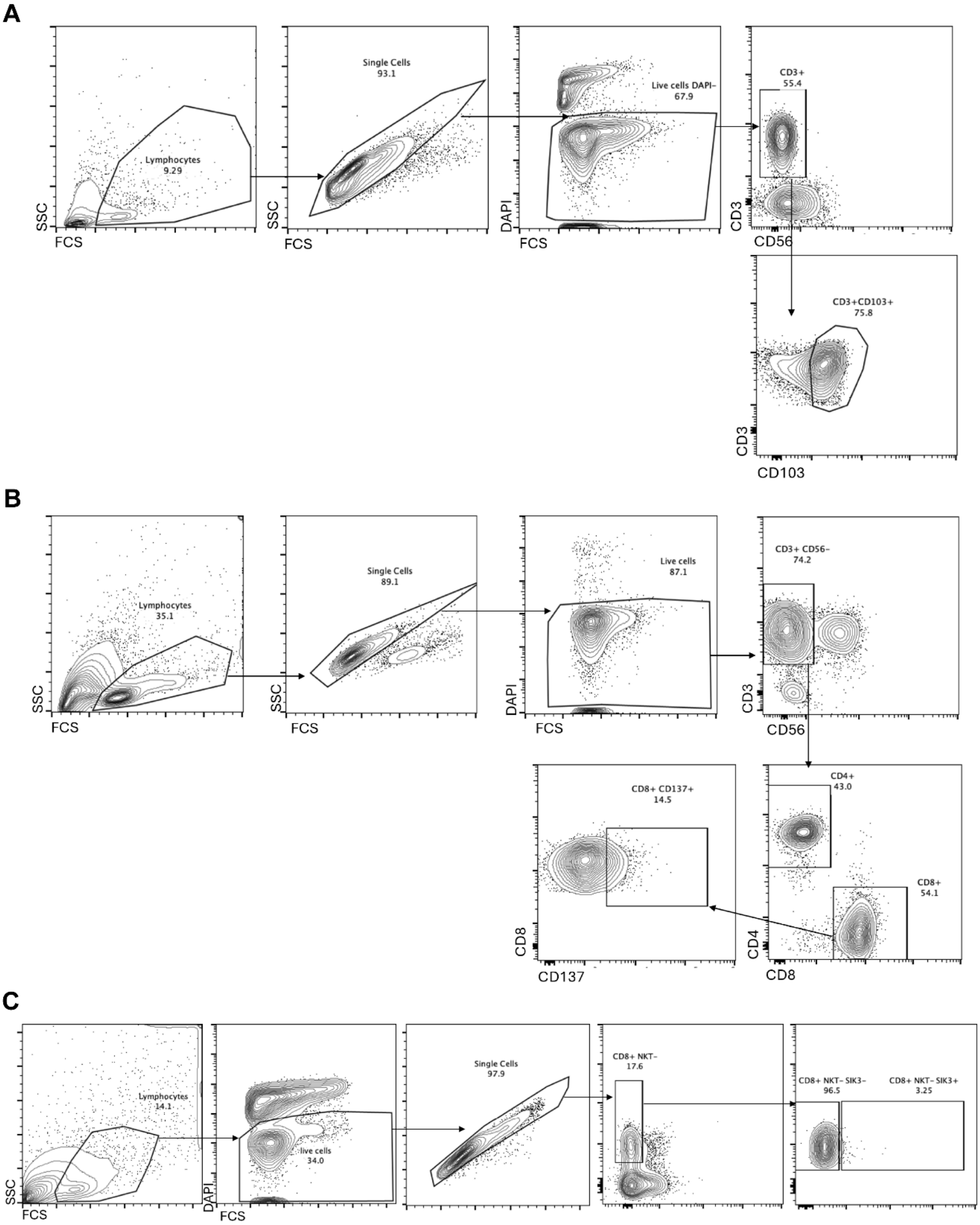

Supplementary Figure 1 Gating strategies used for flow cytometry.

(**A**) Gating strategy to quantify and sort TRMLs (CD3+CD56-CD103+) from paired FT and tumor samples, as assessed by flow cytometry, or CD3+ CD56- from patient PBMCs presented on Fig. 1B and C. used to sort TRMLs for TCR sequencing. (**B**) Gating strategy to sort reactive T cells (CD3 CD56- CD8+ CD137+), from FT TRMLs cocultured with organoids following reactivity assay by IFN-γ ELISPOT. The same strategy was used to sort reactive T cells stimulated with peptides for Analysis of T cell clonotypes in response to predicted tumor neoantigens. **(C)** Gating strategy used for intracellular staining and flow cytometry analysis to evaluate the co-expression of CD8, CD103; Tex1: CD8, GZMK, LAG3; T-SIK3: CD8, SIK3, TOX; Tex2: CD8, CXCL13, HAVCR2/TIM-3, CTLA-4, TIGIT, PDCD1/PD-1

and prolif: CD8 Ki67 on CD8+CD56-, or on CD8+CD56- SIK-3+ and CD8+CD56- SIK-3-, used on the *in vitro* CD8 phenotype switch assay.

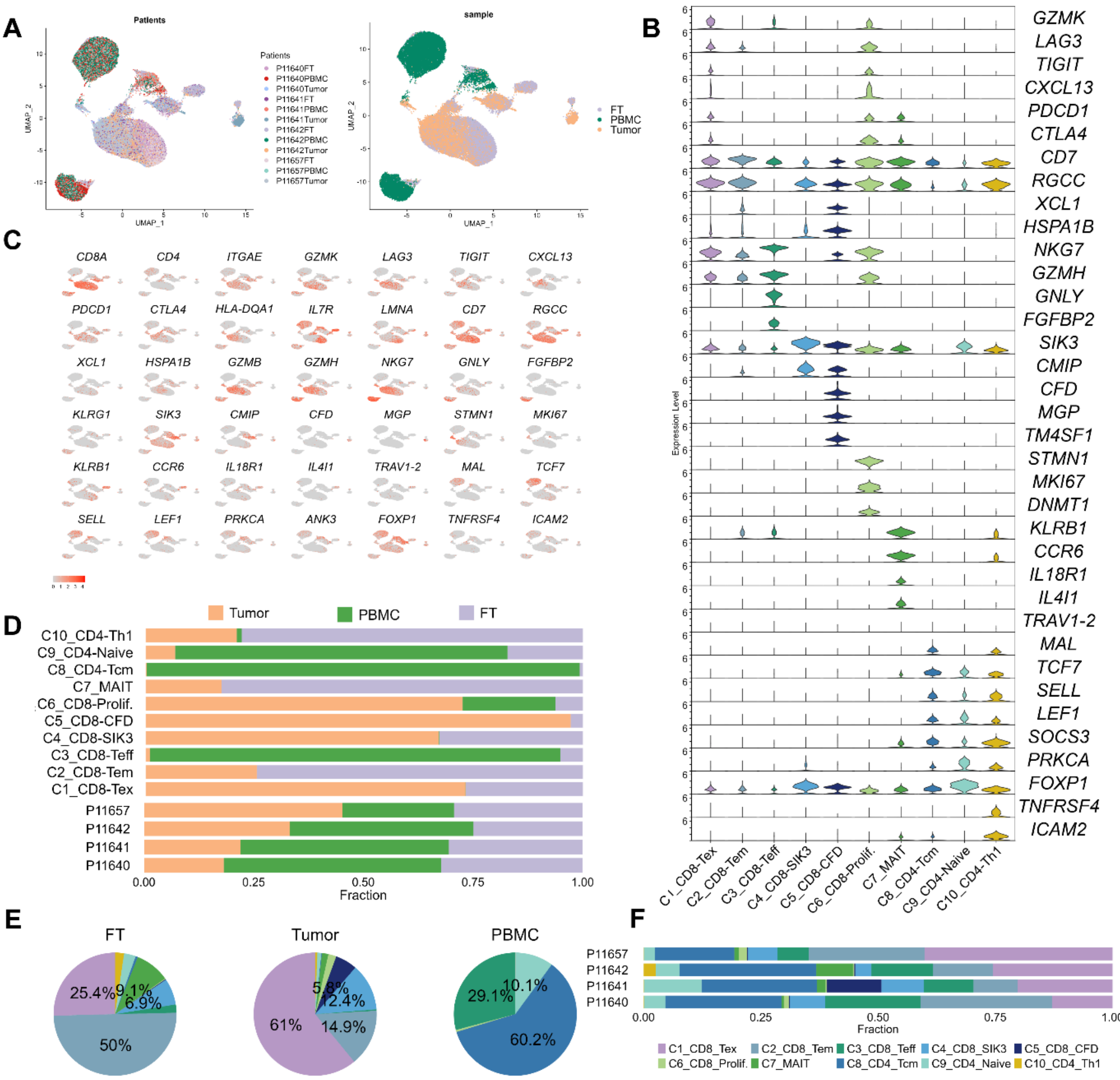

Supplementary Figure 2 Characterization of T cell clusters and their dynamics in HGSOC.

The UMAP plot (A) displays T cells colored by patient origin on the left and by tissue site on the right. The violin plot (B) highlights the expression of canonical T cell subset marker genes, cell subset-specific genes, and key T cell checkpoint genes across the ten identified T cell clusters, with rows representing clusters and columns corresponding to signature genes. The UMAP red-scale projection (C) visualizes the expression levels of these marker and checkpoint genes across different clusters. The bar plots (D) depict the distribution of T cells from each tissue site across clusters at the top, while the bottom section shows the distribution of T cells by patient. The pie chart (E) presents the proportion of T cells within each cluster, categorized by tissue type, including FT, PBMC, and tumor samples. Lastly, bar plots (F) illustrate the cluster distribution of T cells across individual patients, providing insights into inter-patient variability in T cell composition within the HGSOC microenvironment. Source data are provided as a Source Data file.

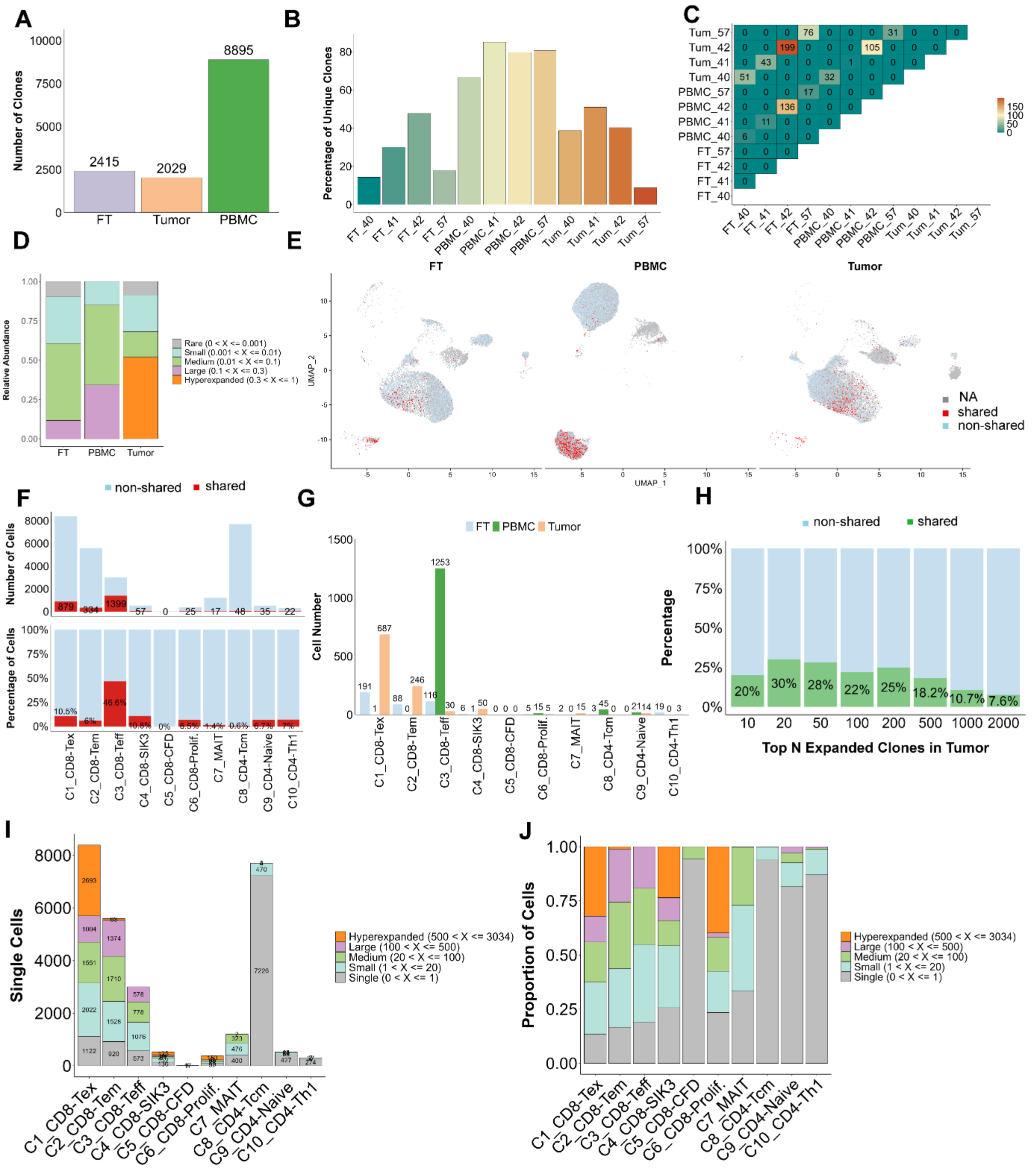

Supplementary Figure 3 Detailed characterization of T cells with shared TCRs between PBMC and tumor tissue in HGSOC patients.

The bar plot (A) illustrates the number of unique TCR clones identified across different tissue sites. The bar plots in (B) depict the distribution of T cell clone sizes in each patient, stratified by tissue site. The clonal overlap plot (C) quantifies the number of shared TCRs between PBMC and tumor for each patient. Clonal abundance across tissue sites is shown in (D), where T cell clones are categorized based on expansion levels: rare (0 < X ≤ 0.001), small (0.001 < X ≤ 0.01), medium (0.01 < X ≤ 0.1), large (0.1 < X ≤ 0.3), and hyperexpanded (0.3 < X ≤ 1), revealing the expansion dynamics of shared clonotypes. The UMAP plot (E) highlights T cells with shared TCRs between tumor and PBMC (red), while non-shared TCRs are marked in light blue, and T cells lacking detected TCRs are in grey. The bar plots (F) display the number and proportion of shared T cells between PBMC and tumor across different clusters. In (G), the shared TCRs across tumor, PBMC, and FT are visualized, showing the number of shared T cells in each cluster and tissue site (FT in blue, PBMC in green, tumor in orange). The bar plot (H)

presents the proportion of shared TCRs between PBMC and the top N expanded clones in tumors, ranging from the top 10 to the top 2000 most expanded clones. These analyses provide insights into the immune dynamics of PBMC-derived T cells and their potential interactions with the tumor microenvironment. Clonal occupancy across clusters is shown in single cell number (I) and proportion (J), where T cell clones are categorized based on clonal expansion: single (n = 1), small (1–20 cells), medium (20–100 cells), large (100–500 cells), and hyperexpanded (500–3034 cells). Source data are provided as a Source Data file.

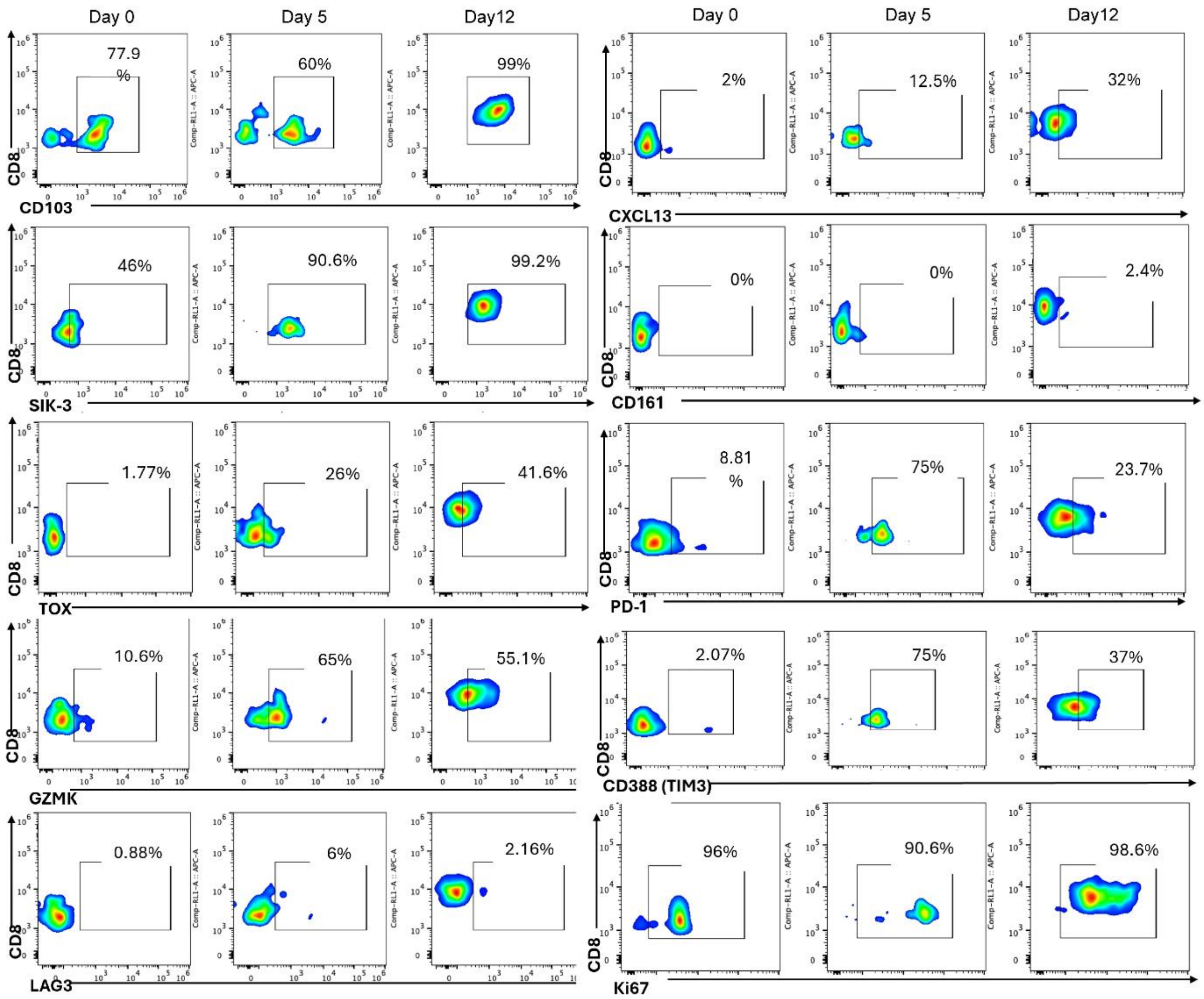

Supplementary Figure 4 Intracellular staining and flow cytometry analysis of CD8 phenotype switching. Flowchart example of intracellular staining and flow cytometry analysis of CD8 phenotype switch. CD8+ T cells from 11699 FT were differentiated with TCR stimulation (anti-CD3/anti-CD28 Dynabeads) and the indicated cytokines for 5 and 12 days. Representative dot plots of different phenotype clusters where quadrants define TRM: CD8, CD103; Tex1: CD8, GZMK, LAG3; T-SIK3: CD8, SIK3, TOX; Tex2: CD8, CXCL13, HAVCR2/TIM-3, CTLA-4, TIGIT, PDCD1/PD-1 and prolif: CD8 Ki67 are shown.

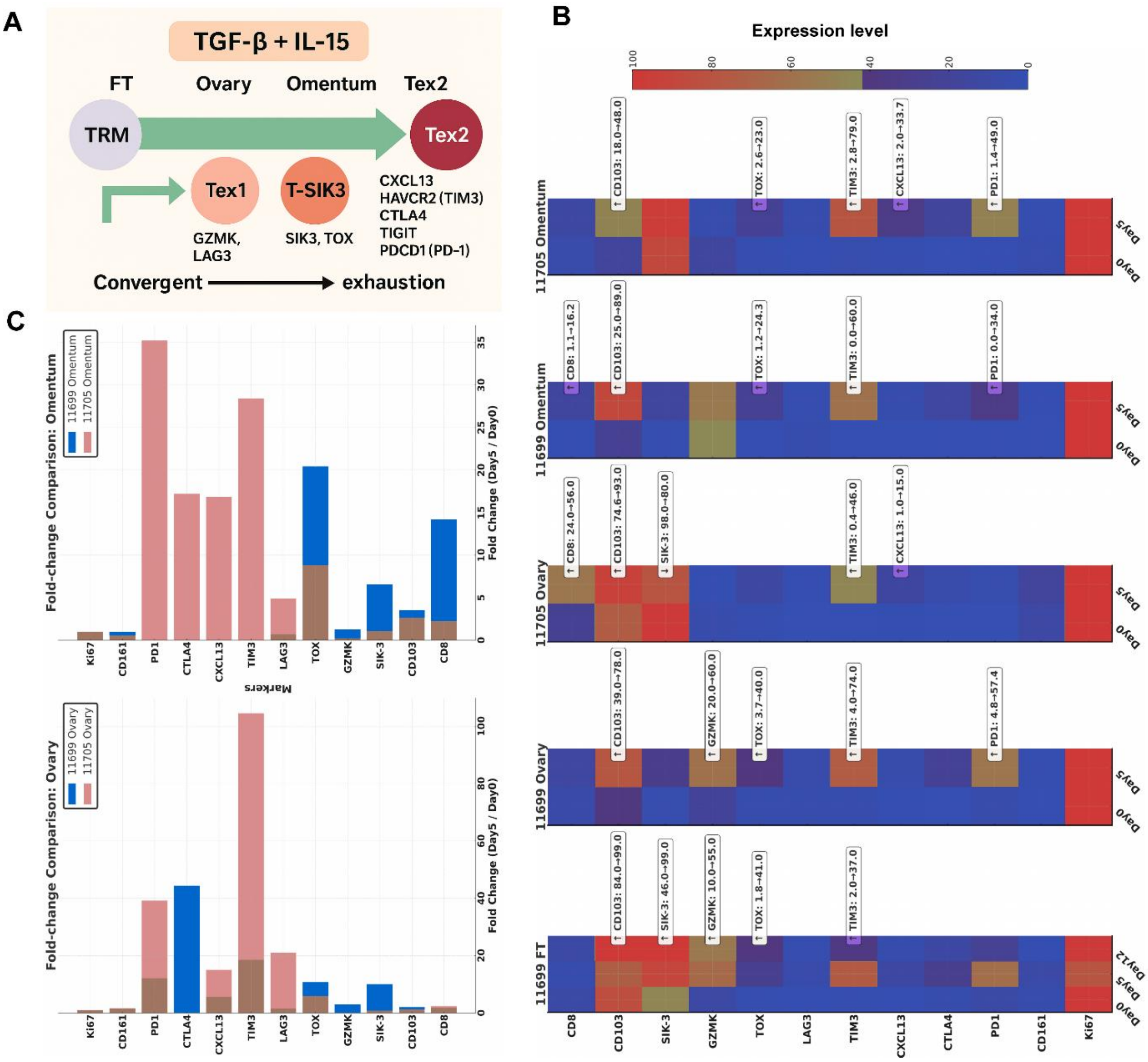

Supplementary Figure 5 CD8⁺ TRMLs phenotype switching in response to TGF-β and IL-15 across tissues and patients.

(A)Diagram summarising the phenotype switch across tissues in response to TGF-β and IL-15 stimulation. (B)Heatmaps show expression changes of key CD8⁺ T-cell markers in samples from two HGSOC patients (IDs 11699 and 11705) collected from the Fallopian Tube, Ovary, and Omentum. Marker levels were measured at baseline (Day 0) and after stimulation (Day 5; Day 12 for 11699 FT) and visualized using a blue-to-red gradient (low to high). Across tissues, CD8⁺ T cells upregulated TRM-associated CD103 and exhaustion-associated markers (TOX, TIM3, CXCL13, CTLA4, PD-1), indicating a coordinated shift toward a TRM-like exhausted phenotype. (C) Fold-change table summarizing quantitative marker differences between patients across tissues. Patient 11705 demonstrates a pronounced collapse of effector-associated GZMK expression and a coordinated rise in inhibitory receptors, whereas patient 11699 displays stronger TRM reinforcement and T-SIK3 pathway activation. Together, the data reveal substantial inter-patient heterogeneity in exhaustion progression despite exposure to identical cytokine stimuli, indicating that the balance between T-SIK3 commitment and Tex2 induction may be patient-specific and tissue-dependent. Source data are provided as a Source Data file.

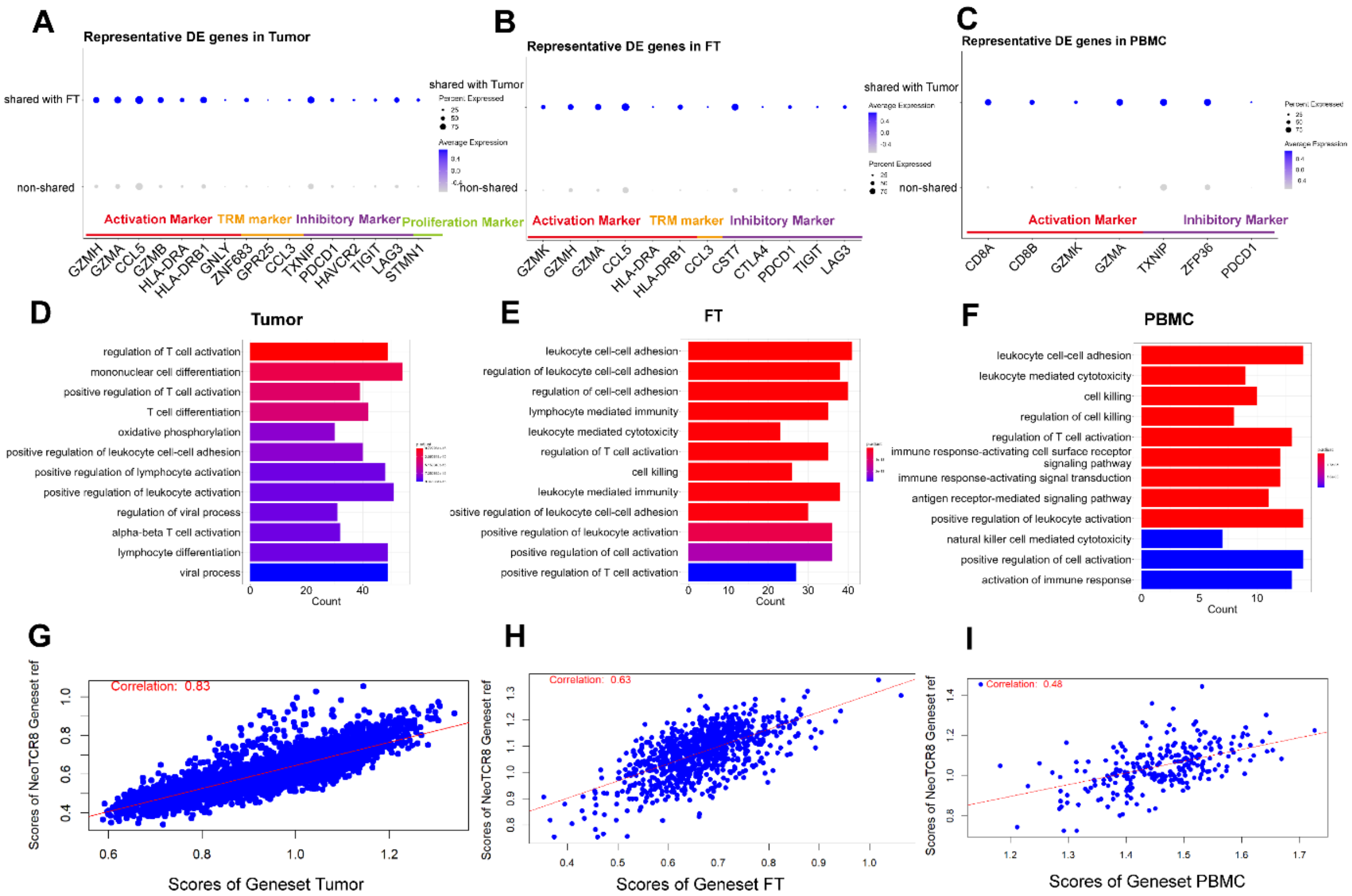

Supplementary Figure 6 The transcriptomic differences between T cells with shared TCRs in tumors and non-shared TCRs across different tissue sites.

(A-C) Dot plots illustrate representative differentially expressed (DE) genes between shared and non-shared TCRs in tumor(A), FT (B), and PBMC (C). (D-F) Gene Ontology (GO) enrichment analyses highlight pathways associated with differentially expressed genes in tumor (D), FT (E), and PBMC (F). (G-I) Correlation plots assess the relationship between DE gene sets from shared versus non-shared TCRs and a published gene set of neoantigen-reactive CD8 T cells (NeoTCR8 Geneset ref.), analyzed separately in tumor (G), FT (H), and PBMC (I) datasets. Source data are provided as a Source Data file.

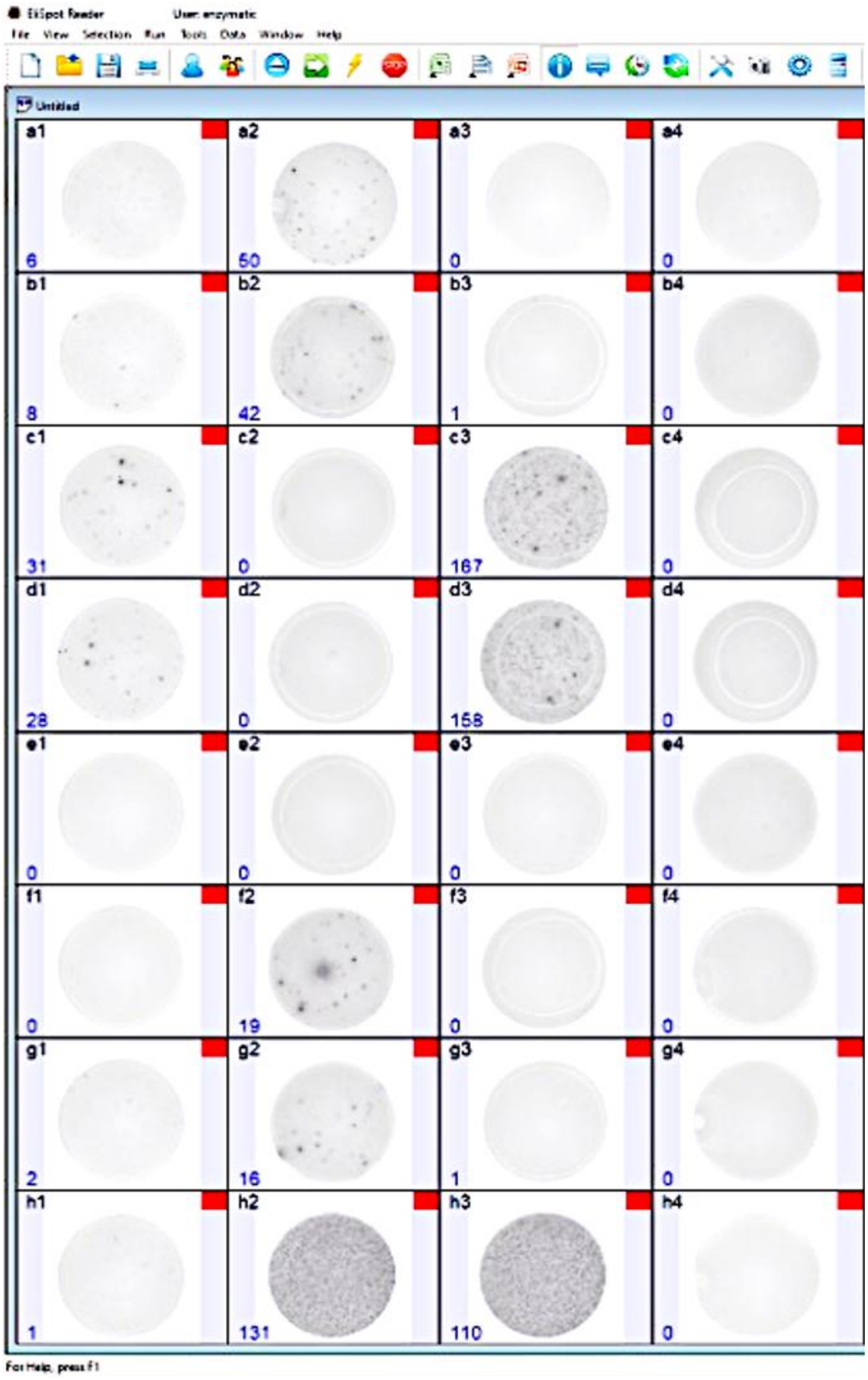

Supplementary Figure 7 IFN-γ ELISPOT analysis of TRML responses following *ex vivo* stimulation.

A representative image of interferon-γ (IFN-γ) ELISPOT wells illustrating the immune response of TRMLs from FT upon *ex vivo* stimulation. Columns 1 and 2 display the ELISPOT results for TRMLs from the FT of patient 11642 following stimulation with medium alone (n=4; a1-b1, a4-b4), tumor organoids (n=4; c1-d1 with 1.5×10^4 TRMLs and a2-b2 with 2×10^5 TRMLs), or FT organoids (n=4; e1, f1, g1, and h1). FT TRMs stimulated with TransAct nanobeads served as a positive assay control (h2). Column 3 shows ELISPOT wells of TRMLs from patient 11639, either unstimulated (a3-b3) or co-cultured with tumor organoids derived from the patient's FT (c3-d3), eliciting a strong IFN-γ response. However, due to the difficulty of identifying normal FT tissue from this patient to establish FT organoids as a control, further comparative analysis was not performed.

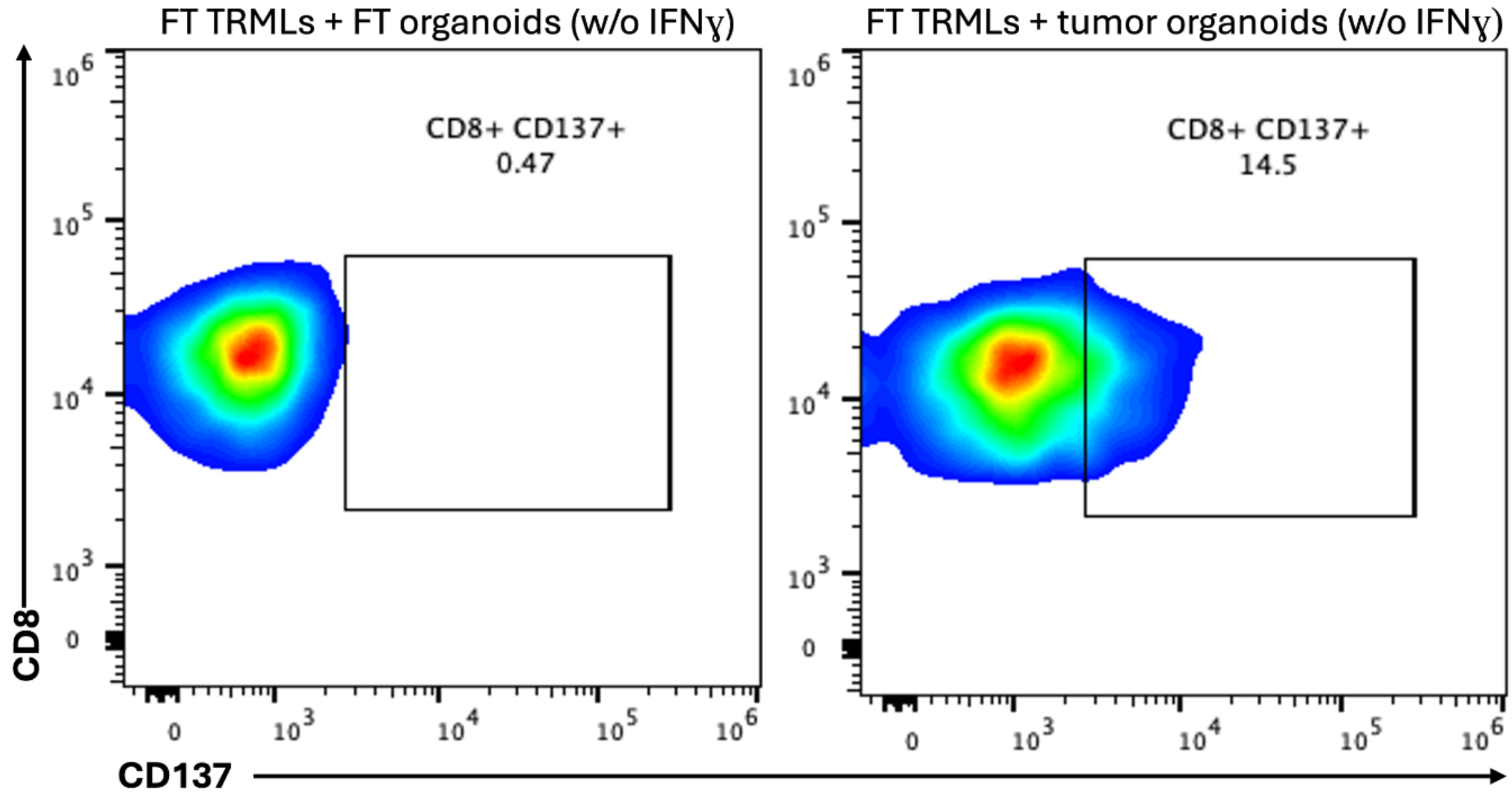

Supplementary Figure 8. Flow cytometry analysis of CD8 and CD137 expression by tumor reactive tissue resident memory T cells (TRML) in FT.
Representative flow cytometry density plots showing CD8 and CD137 expression in TRMLs following coculture with FT organoids or tumor organoids, without prior IFN-γ pre-stimulation.

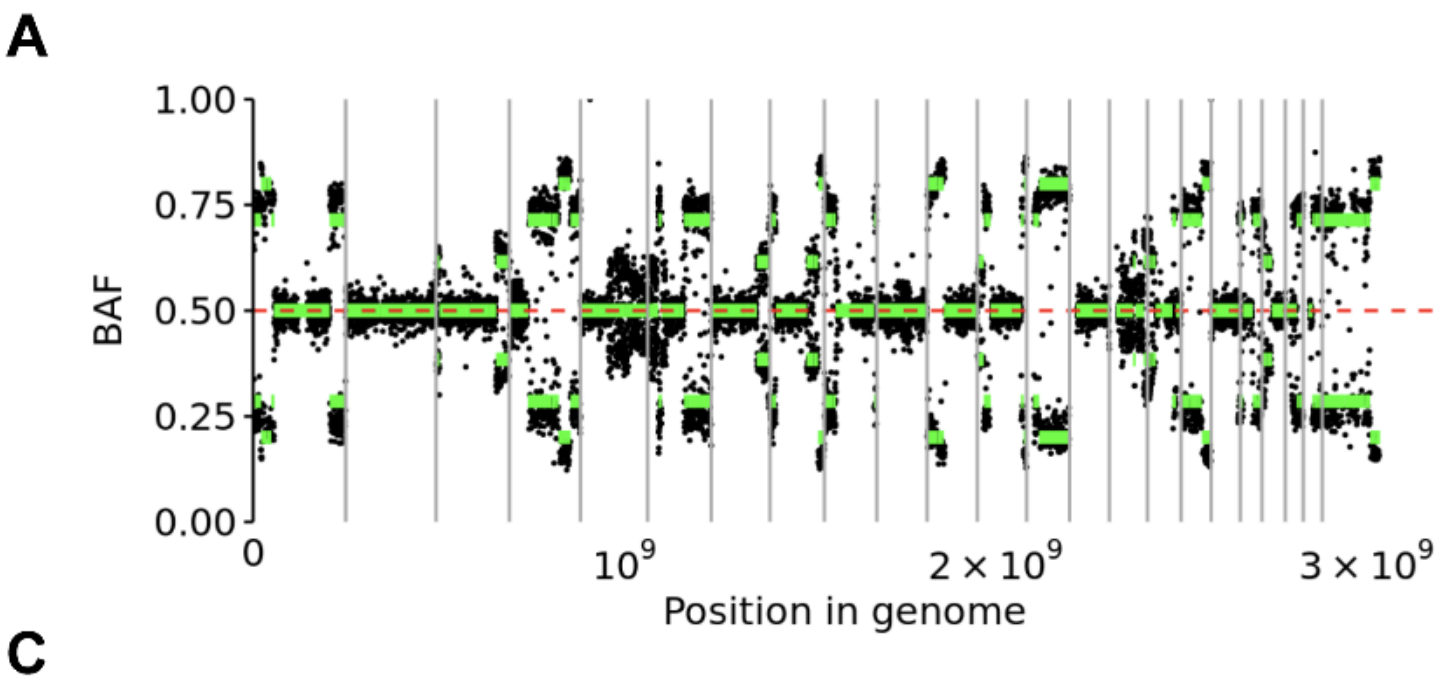

B

Gene	Driver role
BRCA1	TSG
BRCA2	TSG
KRAS	Oncogene
NF1	TSG
PPP2R1A	Oncogene
RB1	TSG
TP53	TSG
ARID1A	TSG
CDK12	TSG

C

Chromosome	Position	Reference	Variant	Gene	Driver role	Consequence	ClinVar
chr17	7578493	C	T	TP53	TSG	Stop-gain	pathogenic
chr17	29556079	C	T	NF1	TSG	Stop-gain	pathogenic
chr17	41243899	AT	A	BRCA1	TSG	Frameshift	

D

	pool 1	pool 2	pool 3	pool 4	pool 5	pool 11
pool 6	MED12_LLLPVLLRY	RBMX_SRGPFQEVL	C4orf21_WLEDVTSGY	PRKAG1_TTTPIYVAL	MED12_LLKLLLPVL	GUCY1A2_LAGVVGERM
pool 7	PRKAG1_VRTTTPIYV	GUCY1A2_ERMPRYCLF	GXYLT1_YADTDILFL	CDC27_YTNMRHLL	CDC27_LPRRSSRLL	MTCH2_KTYCCDLRM
pool 8	TIMP3_SEIVIRAKV	PRKAG1_MVRTTTPIY	LRRC4_VLLSDSGVY	MED12_KLLLPVLLRY	C4orf21_KWLEDVTSGY	
pool 9	GUCY1A2_VVGERMPRY	GUCY1A2_GERMPRYCLF	GXYLT1_LYADTDILF	RBMX_RSRGPFQEV	PRKAG1_AMVRTTTPIY	
pool 10	TIMP3_SEIVIRAKVV	GUCY1A2_ERMPRYCL	GXYLT1_FNSTQVAAM	LRRC4_HVLLSDSGVY	ZMAT1_GTISAHCSL	

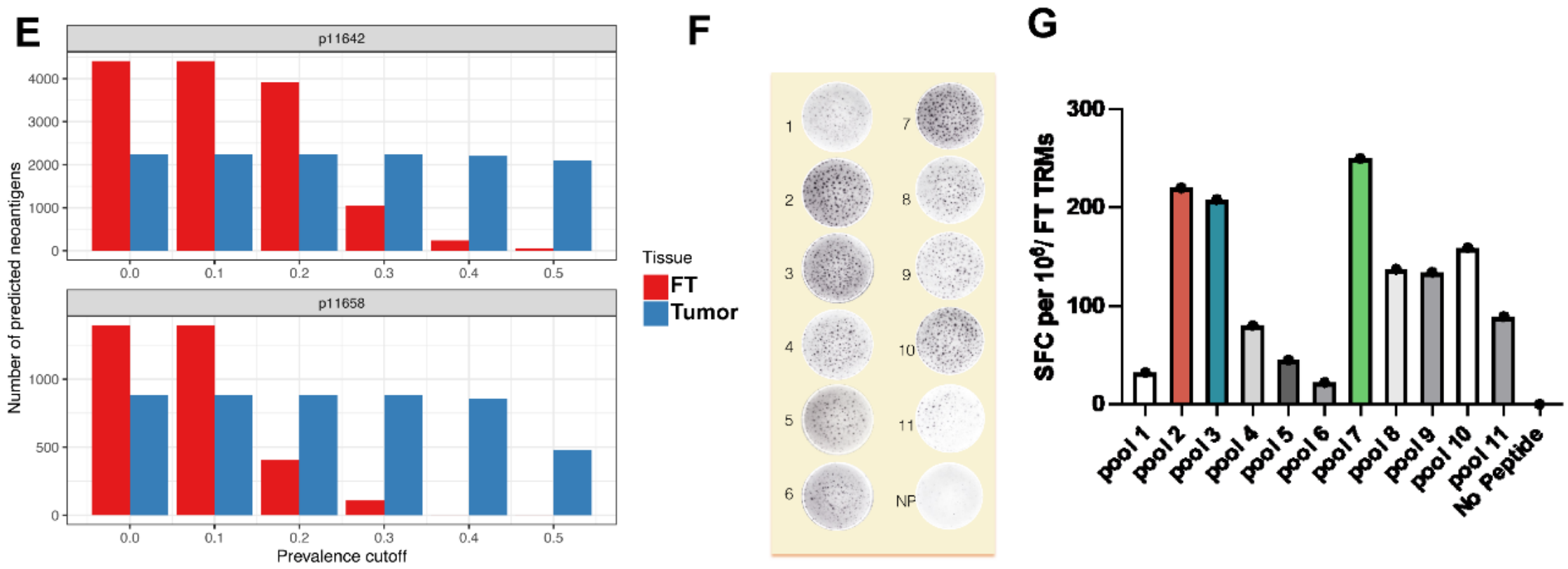

Supplementary Figure 9 Whole-genome sequencing analysis of the tumor, reveals key genomic features of HGSOC.

(A) B allele frequency (BAF) plot showing genome instability, highlighting profound chromosomal alterations characteristic of HGSOC. (B) Copy number variations (CNVs) are illustrated, showing amplifications and deletions across the genome at the site of selected important oncogenes or tumor suppressor genes (TSGs). (C) The presence of driver mutations is identified, providing insights into the molecular mechanisms underlying tumor progression. (D) The peptide pools tested in (E) are detailed in a table listing their individual components. (E) Neoantigen load for FT and tumor across patients at different prevalence levels. (F) Representative ELISPOT spots display IFN-γ responses to eleven pools of 9-mer peptides, indicating immune recognition of potential neoantigens. (G) The number of IFN-γ spot-forming cells (SFCs) per 10^6 FT TRMs is quantified, demonstrating the extent of immune activation. Source data are provided as a Source Data file.

Supplementary Table

Supplementary Table 1: Key resources table

REAGENT OR RESOURCE	SOURCE	IDENTIFIER
Antibodies		
LIVE/DEAD Fixable Violet Dead Cell Stain Kit	Thermo Fisher Scientific	Cat#L3496
DAPI	Miltenyi Biotec Ltd	Cat#130-111-570
Anti-EPCAM, clone 9C4, FITC conjugated	BioLegend	Cat#324210; RRID:AB_756083
Anti-CD3, clone OKT3, BV711 conjugated	BioLegend	Cat#317328; RRID:AB11219592
Anti-CD4, clone A161A1, PE-Cy7 conjugated	BioLegend	Cat#357410; RRID:AB_2565662
Anti-CD8a, clone RPA-T8, APC conjugated	BioLegend	Cat#301014; RRID:AB_2562054
Anti-CD56, clone HCD56, PE conjugated	BioLegend	Cat#318305; RRID:AB_604101
Anti-CD103, clone Ber-ACT8, PerCP/Cyanine5.5 conjugated	BioLegend	Cat#350226; RRID:AB_2716191
BV605-conjugated anti-CD103, clone Ber-ACT8	BioLegend	Cat#350217; RRID:AB_2564282
BV711-conjugated anti-CD56, clone HCD56	BioLegend	Cat#318335; RRID:AB_11218788
BV510-conjugated anti-LAG-3, clone 11C3C65	BioLegend	Cat#369317; RRID:AB_2715780
PerCP-Cy5.5-conjugated anti-GNZK, clone GM26E7	BioLegend	Cat#370513; RRID:AB_2632851
PE-conjugated anti-TOX, clone TXRX10	Thermo Fisher Scientific	Cat#12-6502-82; RRID:AB_10855034
PE-Cy7-conjugated anti-CD161 (KLRB1), clone HP-3G10	BioLegend	Cat#339917; RRID:AB_11126167
PE-Cy7-conjugated anti-Ki-67, clone Ki-67	BioLegend	Cat#350525; RRID:AB_2562871
PE-conjugated anti-CXCL13	BioLegend	Cat#53610; RRID:AB_2941612
BV605-conjugated anti-CD366 (TIM-3), clone F38-2E2	BioLegend	Cat#345017; RRID:AB_2562194
APC-Cy7-conjugated anti-CTLA-4, clone L3D10	BioLegend	Cat#349941; RRID:AB_2922566
BV510-conjugated anti-CD279 (PD-1), clone EH12.1	BioLegend	Cat#563076; RRID:AB_2562255
Anti-CD137, clone 4B4-1, PE conjugated	BioLegend	Cat#309804; RRID:AB_10933428
Anti-CD137 (4-1BB), clone 4B4-1, APC/Cyanine7 conjugated	BioLegend	Cat#309830; RRID:AB_2629644
Mouse anti-human CD28	eBioscience	Cat#16-0289-81; RRID:AB_468926
Anti-human PD1 / nivolumab	Merus / Selleckchem	Cat#A2002; RRID:AB_2810223

FITC-conjugated anti-SIK-3	LSBio	Cat#LS-C458138
Anti-SIK3, polyclonal antibody produced in rabbit	Abcam	Cat#ab110987
Anti-CD8 monoclonal antibody, clone 1033123, mouse	R&D Systems	Cat#MAB3803-SP
Alexa Fluor-conjugated secondary antibodies	Abcam	Cat#ab150074
ProLong™ Gold Antifade Mounting	Thermo Fisher Scientific	Cat#P36930
Human IFN-gamma ELISpotPRO kit (ALP)	Mabtech AB	Cat#3420-2AST-2
TransAct human	Miltenyi Biotec Ltd	Cat#130-111-160
Chemicals, peptides, and recombinant proteins		
RPMI 1640 medium	Gibco	Cat#11875093
Penicillin-Streptomycin (10,000 U/mL)	Gibco Life Technologies	Cat#15140122
gentleMACS C Tubes	Miltenyi Biotec	Cat#130-093-237
Human Tumor Dissociation Kit	Miltenyi Biotec	Cat#130-095-929
ACK lysing buffer	Gibco	Cat#A1049201
Trypan blue solution 0.4%	Thermo Fisher Scientific	Cat#15250061
Matrigel matrix phenol red free mouse EHS tumor	Corning	Cat#356237
Advanced DMEM/F12	Thermo Fisher Scientific	Cat#12634010
HEPES	Thermo Fisher Scientific	Cat#11560496
GlutaMAX-I	Thermo Fisher Scientific	Cat#35050061
B27 Supplement	Thermo Fisher Scientific	Cat#17504044
N2 Supplement	Thermo Fisher Scientific	Cat#17502048
N-acetyl L-Cysteine	Sigma-Aldrich	Cat#A9165-5G
Nicotinamide	Sigma-Aldrich	Cat#N0636-100G
Recombinant human EGF	R&D Systems	Cat#PHG0311
Recombinant human FGF-2	PeproTech	Cat#100-18C
Human Fibroblast Growth Factor-10 (FGF-10)	PeproTech	Cat#AF-100-26
Recombinant human R-Spondin-1	R&D Systems	Cat#120-38
Recombinant human Noggin	PeproTech	Cat#120-10C
A-83-01	Tocris	Cat#2939
Y-27632	Tocris	Cat#72302
Forskolin	Tocris / R&D Systems	Cat#1099/10
DNase I	Roche	Cat#4716728001
Collagenase Type I	Invitrogen	Cat#17018029
DPBS	Thermo Fisher Scientific	Cat#14190144
Trypsin-EDTA (0.5%), no phenol red	Thermo Fisher Scientific	Cat#15400054
IMDM complete medium	Life Technologies	Cat#31980048
Human AB serum	Merck Life Science UK	Cat#H5667-100ML
Interleukin-2 (Proleukin)	Novartis	Not specified
Gentamicin	Lonza / Scientific Laboratory Supplies Limited	Cat#LZ17-518Z
Amphotericin B (Fungizone)	Thermo Fisher Scientific	Cat#15290026
FcR Blocking Reagent, human	Miltenyi Biotec Ltd	Cat#130-059-901

Recombinant human IFN gamma	PeproTech	Cat#300-02
DMSO	Sigma	Cat#sc-358801
Fetal Bovine Serum, heat inactivated	Thermo Fisher Scientific	Cat#10500-064
OKT3 / CD3 Antibody anti-human (OKT3)	Miltenyi Biotec Ltd	Cat#130-093-387; RRID:AB_1036144
Human T-Activator CD3/CD28 for T Cell Expansion and Activation	Thermo Fisher Scientific	Cat#11132D
Recombinant human TGF-β1	PeproTech	Cat#100-21
Recombinant human IL-15	PeproTech	Cat#200-15
Monensin	Sigma-Aldrich	Cat#00-4505-51
Foxp3 Transcription Factor Buffer Set	Sigma-Aldrich	Cat#00-5523
Critical commercial assays		
SpectraMax Quant AccuBlue Pico dsDNA Assay Kit	SpectraMax	Cat#R8355
REPLI-g Advanced DNA Single Cell Kit	Qiagen	Cat#150363
SMARTer Stranded Total RNA-Seq Kit v2 - Pico Input	Takara	Cat#634412
GenElute Single Cell RNA Purification Kit	Sigma-Aldrich	Cat#RNB300
Chromium Next GEM Single Cell 5' Kit v2	10x Genomics	Cat#1000265
Chromium Next GEM Single Cell v2 and V(D)J Reagent Kits	10x Genomics	Not specified
Library Construction Kit	10x Genomics	Cat#1000190
Dual Index Kit TT Set A	10x Genomics	Cat#1000215
Chromium Single Cell Human TCR Amplification Kit	10x Genomics	Cat#1000252
Chromium Next GEM Chip K Single Cell Kit	10x Genomics	Cat#1000287
Software and algorithms		
R 4.2.3	R Core Team	https://www.10xgenomics.com/
Cellranger-7.0.0	10x Genomics	https://www.10xgenomics.com/
Seurat v4.0 (R package)	Satija Lab	https://satijalab.org/seurat/
scRepertoire 2	Borcherding Lab	https://www.borch.dev/uploads/screpertoire/
Monocle 3	Trapnell Lab	https://cole-trapnell-lab.github.io/monocle3/
Harmony (R package)	Immunogenomics Lab	https://github.com/immunogenomics/harmony
