# Supplementary material for: Transcriptomic analysis of tissue-resident memory T cells of the fallopian tube reveals a precursor immune surveillance network for ovarian cancer prevention
Source: Nat Commun. 2026 Jul 13;17:8617. doi: 10.1038/s41467-026-74599-4 (PMC13486501; doi:10.1038/s41467-026-74599-4)
Supplement: Supplementary file 2 [file 41467_2026_74599_MOESM8_ESM.pdf]

nature portfolio

Corresponding author(s): Nancy Zaarour, Ahmed Ashour Ahmed

Last updated by author(s): Jun 25, 2025

Reporting Summary

Nature Portfolio wishes to improve the reproducibility of the work that we publish. This form provides structure for consistency and transparency in reporting. For further information on Nature Portfolio policies, see our Editorial Policies and the Editorial Policy Checklist.

Statistics

For all statistical analyses, confirm that the following items are present in the figure legend, table legend, main text, or Methods section.

n/a	Confirmed	
☐	☒	The exact sample size (*n*) for each experimental group/condition, given as a discrete number and unit of measurement
☐	☒	A statement on whether measurements were taken from distinct samples or whether the same sample was measured repeatedly
☐	☒	The statistical test(s) used AND whether they are one- or two-sided<br>*Only common tests should be described solely by name; describe more complex techniques in the Methods section.*
☒	☐	A description of all covariates tested
☐	☒	A description of any assumptions or corrections, such as tests of normality and adjustment for multiple comparisons
☐	☒	A full description of the statistical parameters including central tendency (e.g. means) or other basic estimates (e.g. regression coefficient) AND variation (e.g. standard deviation) or associated estimates of uncertainty (e.g. confidence intervals)
☐	☒	For null hypothesis testing, the test statistic (e.g. *F*, *t*, *r*) with confidence intervals, effect sizes, degrees of freedom and *P* value noted<br>*Give P values as exact values whenever suitable.*
☒	☐	For Bayesian analysis, information on the choice of priors and Markov chain Monte Carlo settings
☒	☐	For hierarchical and complex designs, identification of the appropriate level for tests and full reporting of outcomes
☒	☐	Estimates of effect sizes (e.g. Cohen's *d*, Pearson's *r*), indicating how they were calculated

Our web collection on statistics for biologists contains articles on many of the points above.

Software and code

Policy information about availability of computer code

Data collection

Single cell RNA sequencing data was collected using the 10X Genomics Chromium Next GEM Single Cell 5' Reagent Kits v2(4 rxns PN-1000265) and Chromium Single Cell Human TCR Amplifcation Kit V(D)J Reagent Kits(16 rxns PN-1000252). 25,000 or 5,000 sequencing reads of 150-bp paired-end per cell for scRNA-seq or VDJ libraries, respectively, were generated on the NovaSeq X Plus instrument. FlowJo (v.10) was used to collect and analyse the flow cytometry data.

Data analysis

This study used established codes, which are available in the following links: 1) Seurat: https://satijalab.org/seurat/ 2)scRepertoire 2 : https://www.borch.dev/uploads/screpertoire/screpertoire/.
Raw sequencing reads from scRNA-seq were processed using Cell Ranger (v7.0, 10X Genomics). Briefly, the base call (BCL) files generated by Illumina sequencers were demultiplexed into fastq files based on the sequences of the sample index and aligned against GRCh38 human transcriptome using STAR.83 Cell barcodes and UMIs associated with the aligned reads were subjected to correction and filtering. Filtered gene-barcodes matrices containing only barcodes with UMI counts passing the threshold for cell detection were imported to Seurat v4.2.172 for downstream analysis. We first removed ambient RNA by the default setting of the R library package SoupX_1.6.284, and then removed the doublets by the default setting of the R library package DoubletFinder_2.0.385. Then keep cell barcodes with more than 300 and lower than 6000 genes expressed, lower than 15% UMIs originated from mitochondrial genes, nCount_RNA more than 1000 and lower than the quantile (nCount_RNA,0.97), and lower than 3% hemoglobin gene. This process resulted in 51,855 cells being left. For each sample, standard library size and log-normalization were performed on raw UMI counts using NormalizeData(), and the top 3000 most variable genes were identified by the ‘‘vst’’ method in FindVariableFeatures(). In each study, individual data were further integrated to remove batch effects and integrate data using the RunHarmony function in the harmony library (harmony_1.2.0). Clustering was performed using the FindClusters() function at resolution 0.2 for integrated data (10 clusters). Uniform manifold approximation and projection (UMAP) was used to visualize single-cell gene expression profile and clustering, using the RunUMAP() function in Seurat with default settings. Differential expression analysis was performed

using the FindAllMarkers() function in Seurat with logfc.threshold=0.25, min.pct=0.1, and test.use=''wilcox''.
FlowJo (v.10) was used to collect and analyse the flow cytometry data.
GraphPad Prism software (ver. 10.4.1., GraphPad, USA) or R software (https://www.r-project.org/) were used for statistical calculation and figure presentation.

For manuscripts utilizing custom algorithms or software that are central to the research but not yet described in published literature, software must be made available to editors and reviewers. We strongly encourage code deposition in a community repository (e.g. GitHub). See the Nature Portfolio guidelines for submitting code & software for further information.

Data

Policy information about availability of data

All manuscripts must include a data availability statement. This statement should provide the following information, where applicable:

- Accession codes, unique identifiers, or web links for publicly available datasets
- A description of any restrictions on data availability
- For clinical datasets or third party data, please ensure that the statement adheres to our policy

The raw single-cell RNA-seq and TCR transcriptomic data generated in this study have been deposited in the Gene Expression Omnibus (GEO) database under accession code GSE301378 (https://www.ncbi.nlm.nih.gov/geo/query/acc.cgi?acc=GSE301378).
Raw whole-genome sequencing (WGS) and bulk RNA-seq data have been deposited in the GEO database under accession codes GSE301179 (https://www.ncbi.nlm.nih.gov/geo/query/acc.cgi?acc=GSE301179) and GSE301180 (https://www.ncbi.nlm.nih.gov/geo/query/acc.cgi?acc=GSE301180), respectively.
Supplementary Data files are provided with this paper. Source data are provided with this paper.
Custom code used for data analysis has been deposited in a public GitHub repository: https://github.com/luyaowang1/scRNAseq_analysis_TRM_ovarian_cancer.

Research involving human participants, their data, or biological material

Policy information about studies with human participants or human data. See also policy information about sex, gender (identity/presentation), and sexual orientation and race, ethnicity and racism.

Reporting on sex and gender

Our research solely involves female participants since the nature of the disease only affects women's reproductive organs.

Reporting on race, ethnicity, or other socially relevant groupings

No socially constructed or socially relevant grouping variables (including race or ethnicity) were used in the design, analysis, or interpretation of this study.

Population characteristics

Patient ID: 11640:
1. age; 75
2. The stage; 3c
3. The grade; High grade serous
4. The histopathology type; High grade serous
5. The surgery and residual disease status; Interval debulking surgery after 3 NACT, complete cytoreduction R0
6. The chemotherapy Carboplatin and Taxol 6 cycles.

Patient ID:11641
1. age; 51
2. The stage; 4A
3. The grade; High grade serous
4. The histopathology type; High grade serous
5. The surgery and residual disease status; Interval debulking surgery after 3 cycles of NACT, No residual disease, R0
6. The chemotherapy 6 cycles of carboplatin and taxol

Patient ID:11642:
1. The age; 54
2. The stage; 3C
3. the grade; High-grade serous
4. The histopathology type; High grade serous
5. The surgery and residual disease status; Interval debulking surgery after 4 cycles of chemo, No residual disease R0
6. The chemotherapy: Carboolatin & Taxol total 7 cycles.

Patient ID 11657
1. Age 71,
2. Stage 4a, High Grade Serous Ovarian Carcinoma of tubo-ovarian origin.
3. BRCA gene mutation negative
p53 positive (mutant)
HR proficient
MMR proficient
PAX8 positive diffuse

4. Past and current diagnosis: Current diagnosis: ovarian tissue replaced by tumour
5. Treatment categories: post 4 cycles of Chemotherapy: 2 cycles of Carboplatin paclitaxel and PLD.

Patient ID: 11658
1. The stage; FIGO stage IV high-grade serous ovarian carcinoma
2. The grade; High-grade serous
3. The histopathology type; High-grade serous carcinoma of tubo-ovarian origin
4. Histopathological findings; Likely origin in the right tube and ovary; mutant p53 pattern; chemotherapy response score (CRS) 3 (almost complete response)
5. Lymph node status; Five lymph nodes retrieved, with necrosis and fibro-inflammatory reaction noted in one para-aortic lymph node; no viable carcinoma identified
6. The surgery and treatment status; Interval debulking surgery following neoadjuvant chemotherapy; only scant viable carcinoma remained in the right ovary
7. Germline tests; BRCA2

Patient ID: 11699
1. The stage; FIGO stage IV
2. The grade; High-grade serous
3. The histopathology type; High-grade serous carcinoma of tubo-ovarian origin
4. Histopathological markers; WT1+, p16+, ER 4/8, CK7+, CK20−
5. Germline tests; BRCA status not known
6. The surgery and treatment status; Four cycles of neoadjuvant chemotherapy (NACT) prior to cytoreductive surgery

Patient ID: 11705
1. The stage; FIGO stage IVB
2. The grade; High-grade serous
3. The histopathology type; High-grade serous ovarian carcinoma
4. Histopathological markers; AE1/3+, WT1+, PAX8+, ER (8/8), CK7+, MOC31+, BerEP4+, calretinin−, mutant p53 staining pattern, Napsin A−
5. The surgery and treatment status; Omental biopsy collected in January 2025; four cycles of neoadjuvant chemotherapy (NACT) prior to cytoreductive surgery

Recruitment

All participants involved in this study provided written informed consent prior to sample collection. Fallopian tube (FT) biopsies for single-cell RNA-seq were collected from the distal fimbrial region of the fallopian tubes. In total, matched blood, FT, and omental tumor samples were collected from four patients with high-grade serous ovarian cancer (HGSOC) for single-cell analyses. Additional patient samples were included in validation and functional experiments.As this study was based on clinically available patient samples, selection bias related to sample availability and suitability for downstream analyses may be present and may limit the generalizability of the findings.

Ethics oversight

Participants in this study were recruited under the Gynecological Oncology Targeted Therapy Study 01 (GO-Target-01), approved by the NRES Committee South Central-Berkshire (approval number: 11/SC/0014).

Note that full information on the approval of the study protocol must also be provided in the manuscript.

Field-specific reporting

Please select the one below that is the best fit for your research. If you are not sure, read the appropriate sections before making your selection.

☒ Life sciences ☐ Behavioural & social sciences ☐ Ecological, evolutionary & environmental sciences

For a reference copy of the document with all sections, see nature.com/documents/nr-reporting-summary-flat.pdf

Life sciences study design

All studies must disclose on these points even when the disclosure is negative.

Sample size

No statistical method was used to predetermine sample size. Sample sizes were determined based on the availability of clinically annotated patient samples and the feasibility of generating matched experimental materials, including organoids. For single-cell RNA-seq and scTCR-seq analyses, four matched FT, tumor, and PBMC samples were included. Validation and functional experiments were performed using all available suitable patient samples. The observed findings were reproducible and consistent across independent experiments and validation assays.

Data exclusions	No samples or datasets were excluded from the study.
Replication	Key findings were reproducible across independent patient samples, validation experiments, and complementary analytical approaches where applicable.
Randomization	Participants and samples were not randomized, as samples were collected based on clinically available matched patient tissues and grouped according to tissue origin and experimental design.
Blinding	Blinding was not formally implemented during data collection and analysis, as sample identities were inherent to the study design and required for downstream analyses.

Behavioural & social sciences study design

All studies must disclose on these points even when the disclosure is negative.

Study description	*Briefly describe the study type including whether data are quantitative, qualitative, or mixed-methods (e.g. qualitative cross-sectional, quantitative experimental, mixed-methods case study).*
Research sample	*State the research sample (e.g. Harvard university undergraduates, villagers in rural India) and provide relevant demographic information (e.g. age, sex) and indicate whether the sample is representative. Provide a rationale for the study sample chosen. For studies involving existing datasets, please describe the dataset and source.*
Sampling strategy	*Describe the sampling procedure (e.g. random, snowball, stratified, convenience). Describe the statistical methods that were used to predetermine sample size OR if no sample-size calculation was performed, describe how sample sizes were chosen and provide a rationale for why these sample sizes are sufficient. For qualitative data, please indicate whether data saturation was considered, and what criteria were used to decide that no further sampling was needed.*
Data collection	*Provide details about the data collection procedure, including the instruments or devices used to record the data (e.g. pen and paper, computer, eye tracker, video or audio equipment) whether anyone was present besides the participant(s) and the researcher, and whether the researcher was blind to experimental condition and/or the study hypothesis during data collection.*
Timing	*Indicate the start and stop dates of data collection. If there is a gap between collection periods, state the dates for each sample cohort.*
Data exclusions	*If no data were excluded from the analyses, state so OR if data were excluded, provide the exact number of exclusions and the rationale behind them, indicating whether exclusion criteria were pre-established.*
Non-participation	*State how many participants dropped out/declined participation and the reason(s) given OR provide response rate OR state that no participants dropped out/declined participation.*
Randomization	*If participants were not allocated into experimental groups, state so OR describe how participants were allocated to groups, and if allocation was not random, describe how covariates were controlled.*

Ecological, evolutionary & environmental sciences study design

All studies must disclose on these points even when the disclosure is negative.

Study description	*Briefly describe the study. For quantitative data include treatment factors and interactions, design structure (e.g. factorial, nested, hierarchical), nature and number of experimental units and replicates.*
Research sample	*Describe the research sample (e.g. a group of tagged Passer domesticus, all Stenocereus thurberi within Organ Pipe Cactus National Monument), and provide a rationale for the sample choice. When relevant, describe the organism taxa, source, sex, age range and any manipulations. State what population the sample is meant to represent when applicable. For studies involving existing datasets, describe the data and its source.*
Sampling strategy	*Note the sampling procedure. Describe the statistical methods that were used to predetermine sample size OR if no sample-size calculation was performed, describe how sample sizes were chosen and provide a rationale for why these sample sizes are sufficient.*
Data collection	*Describe the data collection procedure, including who recorded the data and how.*
Timing and spatial scale	*Indicate the start and stop dates of data collection, noting the frequency and periodicity of sampling and providing a rationale for these choices. If there is a gap between collection periods, state the dates for each sample cohort. Specify the spatial scale from which the data are taken*
Data exclusions	*If no data were excluded from the analyses, state so OR if data were excluded, describe the exclusions and the rationale behind them, indicating whether exclusion criteria were pre-established.*
Reproducibility	*Describe the measures taken to verify the reproducibility of experimental findings. For each experiment, note whether any attempts to repeat the experiment failed OR state that all attempts to repeat the experiment were successful.*
Randomization	*Describe how samples/organisms/participants were allocated into groups. If allocation was not random, describe how covariates were*

	controlled. If this is not relevant to your study, explain why.
Blinding	*Describe the extent of blinding used during data acquisition and analysis. If blinding was not possible, describe why OR explain why blinding was not relevant to your study.*

Did the study involve field work? ☐ Yes ☐ No

Field work, collection and transport

Field conditions	*Describe the study conditions for field work, providing relevant parameters (e.g. temperature, rainfall).*
Location	*State the location of the sampling or experiment, providing relevant parameters (e.g. latitude and longitude, elevation, water depth).*
Access & import/export	*Describe the efforts you have made to access habitats and to collect and import/export your samples in a responsible manner and in compliance with local, national and international laws, noting any permits that were obtained (give the name of the issuing authority, the date of issue, and any identifying information).*
Disturbance	*Describe any disturbance caused by the study and how it was minimized.*

Reporting for specific materials, systems and methods

We require information from authors about some types of materials, experimental systems and methods used in many studies. Here, indicate whether each material, system or method listed is relevant to your study. If you are not sure if a list item applies to your research, read the appropriate section before selecting a response.

Materials & experimental systems

n/a	Involved in the study	
☐	☒	Antibodies
☒	☐	Eukaryotic cell lines
☒	☐	Palaeontology and archaeology
☒	☐	Animals and other organisms
☒	☐	Clinical data
☒	☐	Dual use research of concern
☒	☐	Plants

Methods

n/a	Involved in the study	
☒	☐	ChIP-seq
☐	☒	Flow cytometry
☒	☐	MRI-based neuroimaging

Antibodies

Antibodies used

For flow cytometry:
Anti-human EpCAM-FITC (dilution 1:100, BioLegend, 324210, Clone 9C4)
Anti-human CD103-BV605 (dilution 1:20, BioLegend, 350217, Clone Ber-ACT8)
Anti-human CD3-BV711 (dilution 1:100, BioLegend, 317328, Clone OKT3)
Anti-human CD4-PE-Cy7, (dilution 1:100, BioLegend, 357410, Clone A161A1)
Anti-human CD8-APC (dilution 1:100, BioLegend, 301014, Clone RPA-T8)
Anti-human CD56-BV711 (dilution 1:20, BioLegend, 318335, Clone HCD56)
Anti-human LAG-3-BV510 (dilution 1:20, BioLegend, 369317, Clone 11C3C65)
Anti-human Granzyme K-PerCP-Cy5.5 (dilution 1:20, BioLegend, 370513, Clone GM26E7)
Anti-human TOX-PE (0.5 µg/test, Thermo Fisher Scientific, 12-6502-82, Clone TXRX10)
Anti-human CD161-PE-Cy7 (dilution 1:20, BioLegend, 339917, Clone HP-3G10)
Anti-human Ki-67-PE-Cy7 (dilution 1:20, BioLegend, 350525, Clone Ki-67)
Anti-human TIM-3 (CD366)-BV605 (dilution 1:20, BioLegend, 345017, Clone F38-2E2)
Anti-human CTLA-4-APC-Cy7 (dilution 1:20, BioLegend, 349941, Clone L3D10)
Anti-human PD-1 (CD279)-BV510 (dilution 1:20, BioLegend, 563076, Clone EH12.1)
Anti-human SIK-3-FITC (dilution 1:25, LSBio, LS-C458138)
Anti-CD137 (4-1BB)-APC/Cyanine7 (dilution 1:100, BioLegend, 309830, Clone 4B4-1)

For Immunofluorescence:
Anti-human SIK3 (rabbit) (dilution 1:200, Abcam, ab110987)
Anti-human CD8 (mouse) (dilution 1:200, R&D Systems, MAB3803-SP, Clone 1033123)

For coculture experiments:
CD3 Antibody, anti-human, (OKT3) (dilution: 30ng/ml, Miltenyi Biotec, 130-093-387, Clone: OKT3)
Anti-human PD1 / nivolumab (dilution: 20µg/ml, Merus/Selleckchem, A2002, unknown clone)
Anti-human CD28 (Mouse) (dilution: 5µg/ml, eBioscience, 16-0289-81, Clone CD28)

Validation

1. EpCAM-FITC (BioLegend, 324210, Clone 9C4)
https://www.citeab.com/antibodies/523078-324204-fitc-anti-human-cd326-epcam-antibody
https://www.biolegend.com/en-gb/products/alexa-fluor-488-anti-human-cd326-epcam-antibody-3759
2. CD103-BV605 (BioLegend, 350217, Clone Ber-ACT8)
https://www.biolegend.com/en-ie/products/brilliant-violet-605-anti-human-cd103-integrin-alphae-antibody-10362
https://pubmed.ncbi.nlm.nih.gov/37236191/
3. CD3 (BioLegend, 317328, Clone OKT3)
4. CD4-PE-Cy7 (BioLegend, 357410, Clone A161A1)
https://punchout.biolegend.com/de-de/explore-new-products/pe-cyanine7-anti-human-cd4-antibody-11945
https://jitc.bmj.com/content/9/5/e002222
5. CD8-APC (BioLegend, 301014, Clone RPA-T8)
https://www.biolegend.com/en-gb/products/apc-anti-human-cd8a-antibody-831
https://pubmed.ncbi.nlm.nih.gov/37175900/
6. CD56-BV711 (BioLegend, 318335, Clone HCD56)
https://www.biolegend.com/de-de/products/brilliant-violet-711-anti-human-cd56-ncam-antibody-7943
https://pubmed.ncbi.nlm.nih.gov/33303575/
7. LAG-3-BV510 (BioLegend, 369317, Clone 11C3C65)
https://www.biolegend.com/en-gb/products/brilliant-violet-510-anti-human-cd223-lag-3-antibody-14805
https://pubmed.ncbi.nlm.nih.gov/25941596/
8. Granzyme K-PerCP-Cy5.5 (BioLegend, 370513, Clone GM26E7)
https://www.biolegend.com/en-gb/products/percp-cyanine5-5-anti-human-granzyme-k-antibody-13855
https://ashpublications.org/blood/article/126/8/e1/34746/Live-cell-evaluation-of-granzyme-delivery-and
9. TOX-PE (Thermo Fisher Scientific, 12-6502-82, Clone TXRX10)
https://www.thermofisher.com/antibody/product/TOX-Antibody-clone-TXRX10-Monoclonal/12-6502-82
https://pubmed.ncbi.nlm.nih.gov/38501978/
10. CD161-PE-Cy7 (BioLegend, 339917, Clone HP-3G10)
https://www.biolegend.com/en-gb/products/pe-cyanine7-anti-human-cd161-antibody-7692
https://pubmed.ncbi.nlm.nih.gov/36811599/
11. Ki-67-PE-Cy7 (BioLegend, 350525, Clone Ki-67)
https://www.biolegend.com/en-gb/products/pe-cyanine7-anti-human-ki-67-antibody-9084
https://pubmed.ncbi.nlm.nih.gov/33948570/
12. TIM-3 (CD366)-BV605 (BioLegend, 345017, Clone F38-2E2)
https://www.biolegend.com/en-ie/clone-search/brilliant-violet-605-anti-human-cd366-tim-3-antibody-8606
https://pubmed.ncbi.nlm.nih.gov/36928818/
13. CTLA-4-APC-Cy7 (BioLegend, 349941, Clone L3D10)
https://www.biolegend.com/en-gb/products/apc-cyanine7-anti-human-cd152-ctla-4-antibody-22231?GroupID=BLG10049
https://pubmed.ncbi.nlm.nih.gov/16037385/
14. CXCL13-PE (R&D Systems/Bio-Techne, IC801P, Clone 53610)
https://resources.rndsystems.com/pdfs/datasheets/ic801p.pdf

15. PD-1 (CD279)-BV510 (BD Biosciences, 563076, Clone EH12.1)
https://www.bdbiosciences.com/en-gb/products/reagents/flow-cytometry-reagents/research-reagents/single-color-antibodies-ruo/bv510-mouse-anti-human-cd279-pd-1.563076
https://www.citeab.com/publication/41558351-41869485-efficiency-of-immunological-blood-biomarkers-in-pre
16. SIK-3-FITC (LSBio, LS-C458138)
https://www.lsbio.com/antibodies/sik3-antibody-fitc-conjugated-ls-c458138/458138
17. SIK3 (rabbit) (Abcam, ab110987)
https://www.abcam.com/en-us/products/primary-antibodies/sik3-antibody-ab110987
https://pmc.ncbi.nlm.nih.gov/articles/PMC4222365/
http://dx.doi.org/10.1093/hmg/ddu346
https://aacrjournals.org/cancerdiscovery/article/9/11/1590/10599/An-LKB1-SIK-Axis-Suppresses-Lung-Tumor-Growth-and
https://pmc.ncbi.nlm.nih.gov/articles/PMC6825558/
18. CD8 (mouse) (R&D Systems, MAB3803-SP, Clone 1033123)
https://www.rndsystems.com/products/human-cd8-alpha-antibody_mab3803
https://www.citeab.com/antibodies/1378758-mab3803-cd8-alpha-antibody-clone-1033123
19. Anti-CD137 (4-1BB)-APC/Cyanine7 (BioLegend, 309830, Clone 4B4-1)
RRID: AB_2629644
https://www.biolegend.com/nl-nl/clone-search/apc-cyanine7-anti-human-cd137-4-1bb-antibody-13508
https://www.tandfonline.com/doi/full/10.2217/fon-2020-1013
20. CD28 (mouse) (eBioscience, 16-0289-81, Clone CD28)
21. Anti-PD1 / Nivolumab (Merus/Selleckchem, A2002, clone unknown)
https://www.citeab.com/antibodies/10336182-a2002-nivolumab-anti-pd-1
22. CD3 Antibody (Miltenyi Biotec, 130-093-387, Clone OKT3)
https://www.miltenyibiotec.com/GB-en/products/cd3-antibody-anti-human-okt3.html#conjugate=biotin-functional-grade:size=100-ug-in-1-ml

Eukaryotic cell lines

Policy information about cell lines and Sex and Gender in Research

Cell line source(s) — *State the source of each cell line used and the sex of all primary cell lines and cells derived from human participants or vertebrate models.*

Authentication — *Describe the authentication procedures for each cell line used OR declare that none of the cell lines used were authenticated.*

Mycoplasma contamination — *Confirm that all cell lines tested negative for mycoplasma contamination OR describe the results of the testing for mycoplasma contamination OR declare that the cell lines were not tested for mycoplasma contamination.*

Commonly misidentified lines (See ICLAC register) — *Name any commonly misidentified cell lines used in the study and provide a rationale for their use.*

Palaeontology and Archaeology

Specimen provenance — *Provide provenance information for specimens and describe permits that were obtained for the work (including the name of the issuing authority, the date of issue, and any identifying information). Permits should encompass collection and, where applicable, export.*

Specimen deposition — *Indicate where the specimens have been deposited to permit free access by other researchers.*

Dating methods — *If new dates are provided, describe how they were obtained (e.g. collection, storage, sample pretreatment and measurement), where they were obtained (i.e. lab name), the calibration program and the protocol for quality assurance OR state that no new dates are provided.*

☐ Tick this box to confirm that the raw and calibrated dates are available in the paper or in Supplementary Information.

Ethics oversight — *Identify the organization(s) that approved or provided guidance on the study protocol, OR state that no ethical approval or guidance was required and explain why not.*

Note that full information on the approval of the study protocol must also be provided in the manuscript.

Animals and other research organisms

Policy information about studies involving animals; ARRIVE guidelines recommended for reporting animal research, and Sex and Gender in Research

Laboratory animals	*For laboratory animals, report species, strain and age OR state that the study did not involve laboratory animals.*
Wild animals	*Provide details on animals observed in or captured in the field; report species and age where possible. Describe how animals were caught and transported and what happened to captive animals after the study (if killed, explain why and describe method; if released, say where and when) OR state that the study did not involve wild animals.*
Reporting on sex	*Indicate if findings apply to only one sex; describe whether sex was considered in study design, methods used for assigning sex. Provide data disaggregated for sex where this information has been collected in the source data as appropriate; provide overall numbers in this Reporting Summary. Please state if this information has not been collected. Report sex-based analyses where performed, justify reasons for lack of sex-based analysis.*
Field-collected samples	*For laboratory work with field-collected samples, describe all relevant parameters such as housing, maintenance, temperature, photoperiod and end-of-experiment protocol OR state that the study did not involve samples collected from the field.*
Ethics oversight	*Identify the organization(s) that approved or provided guidance on the study protocol, OR state that no ethical approval or guidance was required and explain why not.*

Note that full information on the approval of the study protocol must also be provided in the manuscript.

Clinical data

Policy information about clinical studies

All manuscripts should comply with the ICMJE guidelines for publication of clinical research and a completed CONSORT checklist must be included with all submissions.

Clinical trial registration	*Provide the trial registration number from ClinicalTrials.gov or an equivalent agency.*
Study protocol	*Note where the full trial protocol can be accessed OR if not available, explain why.*
Data collection	*Describe the settings and locales of data collection, noting the time periods of recruitment and data collection.*
Outcomes	*Describe how you pre-defined primary and secondary outcome measures and how you assessed these measures.*

Dual use research of concern

Policy information about dual use research of concern

Hazards

Could the accidental, deliberate or reckless misuse of agents or technologies generated in the work, or the application of information presented in the manuscript, pose a threat to:

No	Yes	
☐	☐	Public health
☐	☐	National security
☐	☐	Crops and/or livestock
☐	☐	Ecosystems
☐	☐	Any other significant area

Experiments of concern

Does the work involve any of these experiments of concern:

No	Yes	
☐	☐	Demonstrate how to render a vaccine ineffective
☐	☐	Confer resistance to therapeutically useful antibiotics or antiviral agents
☐	☐	Enhance the virulence of a pathogen or render a nonpathogen virulent
☐	☐	Increase transmissibility of a pathogen
☐	☐	Alter the host range of a pathogen
☐	☐	Enable evasion of diagnostic/detection modalities
☐	☐	Enable the weaponization of a biological agent or toxin
☐	☐	Any other potentially harmful combination of experiments and agents

Plants

Seed stocks

Report on the source of all seed stocks or other plant material used. If applicable, state the seed stock centre and catalogue number. If plant specimens were collected from the field, describe the collection location, date and sampling procedures.

Novel plant genotypes

Describe the methods by which all novel plant genotypes were produced. This includes those generated by transgenic approaches, gene editing, chemical/radiation-based mutagenesis and hybridization. For transgenic lines, describe the transformation method, the number of independent lines analyzed and the generation upon which experiments were performed. For gene-edited lines, describe the editor used, the endogenous sequence targeted for editing, the targeting guide RNA sequence (if applicable) and how the editor was applied.

Authentication

Describe any authentication procedures for each seed stock used or novel genotype generated. Describe any experiments used to assess the effect of a mutation and, where applicable, how potential secondary effects (e.g. second site T-DNA insertions, mosiacism, off-target gene editing) were examined.

ChIP-seq

Data deposition

☐ Confirm that both raw and final processed data have been deposited in a public database such as GEO.

☐ Confirm that you have deposited or provided access to graph files (e.g. BED files) for the called peaks.

Data access links
May remain private before publication.

For "Initial submission" or "Revised version" documents, provide reviewer access links. For your "Final submission" document, provide a link to the deposited data.

Files in database submission

Provide a list of all files available in the database submission.

Genome browser session
(e.g. UCSC)

Provide a link to an anonymized genome browser session for "Initial submission" and "Revised version" documents only, to enable peer review. Write "no longer applicable" for "Final submission" documents.

Methodology

Replicates

Describe the experimental replicates, specifying number, type and replicate agreement.

Sequencing depth

Describe the sequencing depth for each experiment, providing the total number of reads, uniquely mapped reads, length of reads and whether they were paired- or single-end.

Antibodies

Describe the antibodies used for the ChIP-seq experiments; as applicable, provide supplier name, catalog number, clone name, and lot number.

Peak calling parameters

Specify the command line program and parameters used for read mapping and peak calling, including the ChIP, control and index files used.

Data quality

Describe the methods used to ensure data quality in full detail, including how many peaks are at FDR 5% and above 5-fold enrichment.

Software

Describe the software used to collect and analyze the ChIP-seq data. For custom code that has been deposited into a community repository, provide accession details.

Flow Cytometry

Plots

Confirm that:

- [x] The axis labels state the marker and fluorochrome used (e.g. CD4-FITC).
- [x] The axis scales are clearly visible. Include numbers along axes only for bottom left plot of group (a 'group' is an analysis of identical markers).
- [x] All plots are contour plots with outliers or pseudocolor plots.
- [x] A numerical value for number of cells or percentage (with statistics) is provided.

Methodology

Sample preparation	Following tissue enzymatic dissociation of tissues, single-cell suspensions were subjected to antibody staining with anti-EPCAM, anti-CD56, anti-CD3 and anti-CD103. Cells were purified by fluorescence-activated cell sorting (FACS) on SONY MA400 cell sorter equipped with four lasers (405 nm, 488 nm, 561 nm and 638nm). A 100-µm nozzle running at 70 psi and 90 kHz was used as the setup for each sort session.<br>FlowJo (v.10) was used to collect and analyze the flow cytometry data. Before gating on fluorescence, single cells were gated using forward scatter (FSC-A) and side scatter (SSC-A) (for intact cells) and SSC-W/SSC-H and FSC-W/FSC-H (to ensure that only singlets were sorted). FACS gates were drawn to include only live single cells based on DAPI (Thermo Fisher Scientific). Further gates were drawn to arrive at CD3+CD103+EpCAM− (for CD103+selected samples) or CD3-EpCAM+ cells (for EPCAM+ selected samples). Boundaries between positive and negative cell fractions were drawn based on single-colour stains. An example gating strategy is shown in Supplementary Methods (Figure S1).
Instrument	SONY MA400 cell sorter
Software	FlowJo (v.10)
Cell population abundance	Cell population abundances is shown in figure1B
Gating strategy	Cells were first gated on forward scatter area (FSC-A) versus side scatter area (SSC-A) to exclude debris. Doublets and cell aggregates were subsequently removed by gating on FSC-H versus FSC-A. Dead cells were excluded based on DAPI positivity, and only live singlet cells were included in downstream analyses. For TCR sequencing analyses, TRMs were sorted as CD3 +CD56−CD103+ cells from paired fallopian tube (FT) and tumor samples, whereas peripheral blood T cells were sorted as CD3 +CD56− cells from matched patient PBMCs (Fig. 1B,C). For identification and isolation of reactive T cells following IFNγ ELISPOT reactivity assays, FT TRMLs were cocultured with autologous organoids, and reactive populations were sorted as CD3 +CD56−CD8+CD137+ cells (Figs. 5B). The same gating strategy was applied to isolate peptide-reactive T cells following stimulation with predicted neoantigen peptides for clonotype analysis of tumor neoantigen–specific T cell responses (Fig. 6). For intracellular staining and flow cytometric phenotypic analyses, TRM populations were defined as CD3+CD56−CD8+CD103 + cells. Exhausted-like T cell subsets were identified as follows: Tex1 cells were defined as CD8+GZMK+LAG3+ cells; T-SIK3 cells as CD8+SIK3+TOX+ cells; and Tex2 cells as CD8+CXCL13+HAVCR2(TIM-3)+CTLA-4+TIGIT+PDCD1(PD-1)+ cells. Proliferating cells were defined as CD8+Ki67+ cells. These analyses were performed on total CD8+CD56− T cells, as well as on CD8+CD56 −SIK3+ and CD8+CD56−SIK3− subsets in the in vitro CD8 phenotype-switch assay (Supplementary figures 1,and 4).

- [x] Tick this box to confirm that a figure exemplifying the gating strategy is provided in the Supplementary Information.

Magnetic resonance imaging

Experimental design

Design type	*Indicate task or resting state; event-related or block design.*
Design specifications	*Specify the number of blocks, trials or experimental units per session and/or subject, and specify the length of each trial or block (if trials are blocked) and interval between trials.*
Behavioral performance measures	*State number and/or type of variables recorded (e.g. correct button press, response time) and what statistics were used to establish that the subjects were performing the task as expected (e.g. mean, range, and/or standard deviation across subjects).*

Acquisition

Imaging type(s)	*Specify: functional, structural, diffusion, perfusion.*
Field strength	*Specify in Tesla*
Sequence & imaging parameters	*Specify the pulse sequence type (gradient echo, spin echo, etc.), imaging type (EPI, spiral, etc.), field of view, matrix size, slice thickness, orientation and TE/TR/flip angle.*
Area of acquisition	*State whether a whole brain scan was used OR define the area of acquisition, describing how the region was determined.*

Diffusion MRI ☐ Used ☐ Not used

Preprocessing

Preprocessing software	*Provide detail on software version and revision number and on specific parameters (model/functions, brain extraction, segmentation, smoothing kernel size, etc.).*
Normalization	*If data were normalized/standardized, describe the approach(es): specify linear or non-linear and define image types used for transformation OR indicate that data were not normalized and explain rationale for lack of normalization.*
Normalization template	*Describe the template used for normalization/transformation, specifying subject space or group standardized space (e.g. original Talairach, MNI305, ICBM152) OR indicate that the data were not normalized.*
Noise and artifact removal	*Describe your procedure(s) for artifact and structured noise removal, specifying motion parameters, tissue signals and physiological signals (heart rate, respiration).*
Volume censoring	*Define your software and/or method and criteria for volume censoring, and state the extent of such censoring.*

Statistical modeling & inference

Model type and settings	*Specify type (mass univariate, multivariate, RSA, predictive, etc.) and describe essential details of the model at the first and second levels (e.g. fixed, random or mixed effects; drift or auto-correlation).*
Effect(s) tested	*Define precise effect in terms of the task or stimulus conditions instead of psychological concepts and indicate whether ANOVA or factorial designs were used.*

Specify type of analysis: ☐ Whole brain ☐ ROI-based ☐ Both

Statistic type for inference<br>(See Eklund et al. 2016)	*Specify voxel-wise or cluster-wise and report all relevant parameters for cluster-wise methods.*
Correction	*Describe the type of correction and how it is obtained for multiple comparisons (e.g. FWE, FDR, permutation or Monte Carlo).*

Models & analysis

n/a	Involved in the study
☐	☐ Functional and/or effective connectivity
☐	☐ Graph analysis
☐	☐ Multivariate modeling or predictive analysis

Functional and/or effective connectivity	*Report the measures of dependence used and the model details (e.g. Pearson correlation, partial correlation, mutual information).*
Graph analysis	*Report the dependent variable and connectivity measure, specifying weighted graph or binarized graph, subject- or group-level, and the global and/or node summaries used (e.g. clustering coefficient, efficiency, etc.).*
Multivariate modeling and predictive analysis	*Specify independent variables, features extraction and dimension reduction, model, training and evaluation metrics.*
