# Supplementary material for: Transcriptomic analysis of tissue-resident memory T cells of the fallopian tube reveals a precursor immune surveillance network for ovarian cancer prevention
Source: Nat Commun. 2026 Jul 13;17:8617. doi: 10.1038/s41467-026-74599-4 (PMC13486501; doi:10.1038/s41467-026-74599-4)
Supplement: Supplementary file 3 [file 41467_2026_74599_MOESM9_ESM.pdf]

nature portfolio

Peer Review File

Transcriptomic analysis of tissue-resident memory T cells of the fallopian tube reveals a precursor immune surveillance network for ovarian cancer prevention

Corresponding Author: Professor Ahmed Ahmed

This file contains all reviewer reports in order by version, followed by all author rebuttals in order by version.

Version 0:

Reviewer comments:

Reviewer #1

(Remarks to the Author)
Review Comments

In the manuscript entitle “Single cell functional immunogenomics of the fallopian tube reveals a precursor immune surveillance network for ovarian cancer prevention” Wang et al, performed single-cell RNAseq and TCRseq of T cells from three matched tissue sites of high-grade serious ovarian cancer. The authors observed the presence of resident-memory T cells in the non-cancerous FT and demonstrated a higher TCR overlap between the TRM of non-cancerous FT and the TRM of tumor compared to circulating T cells. Non-cancerous FT-derived TRM recognized autologous tumor antigens and shared neoantigen-reactive T-cell clonotypes with tumor-infiltrating TRM.
Overall, the manuscript contains novel aspects, especially the recognition of neoantigen by the non-cancerous derived TRM, which is supported by strong data. The difference of transcript expression and signature between TRM from FT and tumors is interesting, but not so impactful given the fact that several studies described the exhausted signature in tumor-infiltrating TRM from different cancer types. The TCR shared between T cells from FT and ovarian tumors is well demonstrated, but the description of results is sometimes confusing and lacks some comparison to fully support the conclusion.
The link between the FT TRM and the protection and/or the prevention of cancer is not supported by solid data and is limited to the observation of TRM in the FT from four patients.

Major Comments
Figure 1: It is surprising that any CD4 T regulatory cluster is found at least in the tumor compartment. The author should discuss or explain the absence of Tregs?
The link in the figure between TRM and the overall scRNAseq is not clear. Do the scRNAseq were performed on total T cells from tissues and tumors or only on TRM? Which cell surface markers were used for the sorting of resident T cells? Authors should clarify this technical aspect as there is not TRM annotations on the scRNAseq analysis.
It is surprising that there is no detection of TRM clusters or Trm signature in the scRNAseq analysis and no description of Trm clusters was included in the main text. The authors should clarify in the result section which Trm clusters or T-cell clusters are described.
Do the authors consider the MAIT cluster as TRM cells?
Figure 2: If the scTCRseq was done only on TRM from tissues and T cells from PBMC, it makes difficult to conclude on the expansion comparison or TCR lineage as the T cells that are not TRM could may be also expand in the tumor tissue. The tumor-reactive T cells argument is based only on the sharing TCR is not strongly convincing. If TCR clonotype is shared between clusters, this means that tumor-specific T cells are distributed in all populations? The reading and conclusion of the paragraph describing figure 2 is not easy to follow and the overall message is difficult to catch.

Figure 3: which gene or features distinguish the two cluster of T-ex? What is the rational to split this cluster in two clusters?
Figure 3G: Is the proportion of CD8 SIK3 positive representative of the cluster proportion in the scRNAseq? It would be interesting to validate the phenotype switch of this CD8 T-cell population at the protein levels and/or with in vitro experiments.
Figure 3H: The conclusion on the clonal diversity within the cluster CD8_SIK3 is not clear. What is the main message? Because this cluster is at the end of the pseudotime trajectory, what is the hypothesis about the fact that these cells are not

expanded? Why the percentage of unique clone is even higher in the tumor tissue while these T cells undergo more differentiation and exhaustion switch? How is the heterogeneity on the percent of the cluster and TCR clonality between patients?
Authors claim that CD8_SIK3 cluster and more precisely the shared TCR clonotypes undergo a specific upregulation of some genes depending of the environment. What is the effect of this environment on others cluster? Is T_ex also have a similar activation trend in the tumor compared to FT? What is the effect on non-shared TCR clonotypes? Especially given the fact that most of the TCR of these clusters are unique.
In the same line, the authors should demonstrate that the exhausted and activation features upregulated in tumors, and thus the tumor-reactive signature, is not happening in the non-shared TCR cells (FT vs tumor) to argue that the tumor-reactivity is restricted to shared TCR-expressing cells.

Authors discussed the possibility that SIK3-expressing TRM cells exhibit a transition state of differentiation before exhausted state, however the SIK3-TRM appear at the end of the trajectory analysis, which does not support this hypothesis. Moreover, this cluster seems more present in the tumor tissue compared to FT, in a opposite trajectory compared to T-ex2. Authors should clarify the relationship between SIK3-expressing and T_ex clusters. How is the TCR sharing between these clusters and how it changes in the FT or the tumor?

It would be interesting to estimate the level of neoantigens present in FT and in tumors to explore the impact of antigen expression and chronicity on the differentiation state of TRM.

Minor:
Figure 1: There is a problem of figure panel correspondence in the legend.
Some legend in the figure are too small leading to difficulty to understand the information.
Lack of title or legend on supplementary tables.

Reviewer #2

(Remarks to the Author)
I co-reviewed this manuscript with one of the reviewers who provided the listed reports. This is part of the Nature Communications initiative to facilitate training in peer review and to provide appropriate recognition for Early Career Researchers who co-review manuscripts.

Reviewer #3

(Remarks to the Author)
This carefully constructed manuscript takes a deep dive into T cell dynamics in high-grade serous ovarian cancer (HGSC). This study is addressing a clinically relevant question - despite remarkable genomic instability, these tumors are generally 'immune cold' and don't respond well to PD1/PDL1 checkpoint blockade but we have a poor understanding of the T cell dynamics during the evolution of these tumors. Although this report used a relatively small sample size for initial discovery (4 patients), the team profile multiple tissues across the cohort using single cell sequencing of transcriptomes and TCRs to map clonality and differentiation of T cells in likely tissue of origin (FT), tumors and omental metastases. A number of key findings are reported including a novel SIK3+ T cell state that may represent a distinct exhaustion trajectory in HGSC, and a high degree of sharedness between fallopian T cell TCRs and tumor TCRs reflecting tumor origins. The study is thorough, using orthogonal assays to validate/extend key findings. The new SIK3+ subset is validated by IF and the team also use sophisticated autologous co-culture studies to validate key findings and identify potential neoantigen targets of expanded TCR clones. Overall, this is an important and non-incremental study that sheds light on T cell dynamics in HGSC and could lead to the development of new immunomodulatory therapeutic strategies for these highly lethal tumors. There is great enthusiasm for this report, with a few opportunities for improvement through minor revision, as follows –

1. On the whole the statistical analyses are thorough but in two places the team could consider using more rigorous methods
a. Paragraph starting on 138 – there are tools to compare cell composition and subset abundance (MILO, Speckle and others) that take interpatient variability into account
b. Paragraph starting line 351 – the authors could consider testing if the overall composition of data points are different in FT versus HGSC, rather than contrasting correlations (it may be that the effects go in a similar direction for example, but with a smaller magnitude)
2. Figures:
a. Many of the figures have very small text, which was challenging to read when printed, for accessibility it is recommended to increase the font size throughout
b. On the UMAPs, including the n cells can help the reader to better help the reader contextualize the data and understand the impact of any filtering
c. Figure 5 panel H needs a more detailed figure legend for readers not familiar with this assay/data type
3. A code availability statement was included but there are no details of data availability.

Reviewer #4

(Remarks to the Author)
The article by Wang et al. describes tissue resident memory T-cells (TRMLs) and their hypothesized role within HGSOC

development. The authors show the presence of these TRMLs within (healthy) fallopian tube tissue of HGSOC patients (n=4), and indicate towards their tumor responsiveness and lower exhaustion profile in comparison to TRMLs within omental tumors. However, the impact of these results are limited due to some important considerations which are lacking. For example, the only immune cells that are focused on are CD3+ CD103+ T-cells, while many other immune cell populations have been found to play an important role in HGSOC. Moreover, the conclusions made in this article are still widely based on a lot of assumptions and hypotheses, and should be reevaluated. Overall, this study uncovers a potentially important immune cell population within early development of HGSOC but should be reported differently with more scientific facts and more limited assumptions. Specifically, the article presents these cells as a solution for immunotherapy in these patients, which is a big leap from the findings presented here in a limited patient population. The article should be written more fundamentally.
Some major comments:
- The first assumption lies in the importance of these TRMLs. As STIC lesions are usually non-invasive in early ovarian tumor development, the authors hypothesize that these lesions are contained due to the presence of TRMLs.
o While this may be correct, this should be placed in the wider context. In general, early stage cancer is known to induce an anti-cancer immune response which can often be evaded or escaped by tumor cells when disease progresses. HGSOC patients are often diagnosed at a late stage of the disease in which immune escape and immunosuppression are highly abundant. Herein, many non-lymphoid immune cells play an important role, which is left untouched in this study.
o The TRMLs evaluated in this study are derived from HGSOC patients (during debulking surgery? In what disease stage?). While they are derived from 'non-tumoral fallopian tubes', cancer cells are present within the body of these patients. It should be discussed that the similar TCR expression and tumor recognition by the FT TRMLs can be related to neoantigens/TAAs being present within the peritoneal cavity/ascites of these patients? And can therefore not be extrapolated to the situation in early stage HGSOC?
The results don't directly indicate the anti-tumor effect of these cells in early disease stages, as this is not what is being evaluated here. But this is one of the main conclusions of this paper and should be nuanced more! Moreover, again early stage HGSOC is not often diagnosed so relevance of this is lacking.
o I do agree on the potential importance of these FT TRMLs for adoptive T-cell transfer after surgery due to their lower exhaustion and tumor responsiveness. This is an interesting approach to further explore, but should be placed within the wider immunological environment of HGSOC as T-cells alone may not be enough to overcome the high immunosuppression..

Comments:
- Introduction:
o Would suggest to already indicate to the high immunosuppression in HGSOC TME in the introduction and more background on what is already known immunologically.
- M&M:
o please give more information on the patient population used. The immune landscape can differ greatly in patients depending on their disease stage and previous treatment cycles.
o For the FT, how did you confirm that the biopsy used in your study did not contain tumors? IHC? Can this be added in the supplementals? This is only described in the results rection, please provide details in M&M. Moreover, did you consider comparison of immune landscape in STIC lesions as well?
It seems like a bit of a stretch to assume that the TRMLs that you find in healthy fallopian tube tissue (but derived from cancer patient) are the same cells that contain the invasiveness of STIC lesions without actually evaluating these STIC lesions themselves? Same goes for the TRMLs at early disease stages. How can you be sure that these are similar to the TRMLs that you analyze here which are derived from advanced stage patients?
o Please add the amount of patients and amount of samples used per assay in the M&M and results.
o scRNAseq was only performed on HGSOC tissue? So only on tumor tissue and not FT? be more specific in description of the materials used.
- Results
o Why did you opt to take tumor-free FT and omental tumors? Why not directly compare tumor-free and tumor-bearing fallopian tubes? Can the fact that the tissue is different not explain why there are many more TRMLs in the tumor-free FT compared to the omental tumor? As the omentum has much less epithelial area for the TRMLs to adhere to?
o Line 138, there is no Figure 1F?
o Quality of the figures should be improved. Some legends are not readable.
- Discussion
o It is important to discuss the importance of your findings. For example, results from figure 3, while impressively gathered and interesting, are not very surprising. The fact that exhaustion of immune cells is higher in the tumor region compared to the non-tumoral area seems quite logical, as the non-tumoral area is exposed less to inflammation, cytokines and antigens release.
o Please discuss if there is any indication that these TRMLs are also this abundantly present in earlier stages in the disease, proven in other paper? You have only shown advanced disease.
o Please discuss limited patient population.
o When discussing the potential of these TRMLs for use in vaccinations or preventative therapy, it is important to discuss the relevance of this in HGSOC, which is very rarely found at a stage that preventative measures can still be used..
Focus could be more on the adaptive T-cell transfer therapy as this is more logical and supported more by your results.
o It is indicated in the reporting summary that all patients received NACT, meaning they have received multiple cycles of chemotherapy before debulking surgery in which the samples were taken? Please disclose how the chemotherapy regimen can impact the immune cells.

Version 1:

Reviewer comments:

Reviewer #1

(Remarks to the Author)
Thanks to the authors who have adequately answer to most of the questions and comments.
I only have one last concern about the flow cytometry data. In the scRNAseq analysis, the CD8-SIK3 cluster (Fig 3B) represents a small proportion among TRM cells, which is not confirmed by flow cytometry analysis (Fig S4-5), where a broader proportion of CD8 T cells (or TRM cells?) expressing SIK3 is observed (46% at d0). The authors should comment on this discrepancy?
In the Fig S5 and the flow cytometry analysis, it would be better to see the phenotype changing during stimulation at the T-cell population level, not at each marker individually. What is the exact phenotype switch for the CD8 TRM SIK3 population regarding the exhausted/activation markers compared to the CD8 Tex population? Is the same result observed for TRM and non-TRM populations?

Reviewer #2

(Remarks to the Author)
I co-reviewed this manuscript with one of the reviewers who provided the listed reports. This is part of the Nature Communications initiative to facilitate training in peer review and to provide appropriate recognition for Early Career Researchers who co-review manuscripts.

Reviewer #3

(Remarks to the Author)
The reviewers have done a thorough job of responding to the comments in the initial review. This is an important manuscript and valuable insights for those researching ovarian cancer and fallopian tube biology.

Reviewer #5

(Remarks to the Author)
I assessed the manuscript and response letter focusing on reviewer #4 and whether the authors have addressed the concerns raised by Rew #4.

In my view, the authors have satisfactorily addressed the concerns and added mostly descriptions and clarifications to the manuscript concerning the materials and methods. Further, they have modified the discussion to address the reviewers' comments on the interpretation of the results.

Open Access This Peer Review File is licensed under a Creative Commons Attribution 4.0 International License, which permits use, sharing, adaptation, distribution and reproduction in any medium or format, as long as you give appropriate credit to the original author(s) and the source, provide a link to the Creative Commons license, and indicate if changes were made.
In cases where reviewers are anonymous, credit should be given to 'Anonymous Referee' and the source.
The images or other third party material in this Peer Review File are included in the article's Creative Commons license, unless indicated otherwise in a credit line to the material. If material is not included in the article's Creative Commons license and your intended use is not permitted by statutory regulation or exceeds the permitted use, you will need to obtain permission directly from the copyright holder.
To view a copy of this license, visit https://creativecommons.org/licenses/by/4.0/

RESPONSE TO REVIEWERS' COMMENTS

Reviewer #1 (Remarks to the Author):

Review Comments

In the manuscript entitle "Single cell functional immunogenomics of the fallopian tube reveals a precursor immune surveillance network for ovarian cancer prevention" Wang et al, performed single-cell RNAseq and TCRseq of T cells from three matched tissue sites of high-grade serious ovarian cancer. The authors observed the presence of resident-memory T cells in the non-cancerous FT and demonstrated a higher TCR overlap between the TRM of non-cancerous FT and the TRM of tumor compared to circulating T cells. Non-cancerous FT-derived TRM recognized autologous tumor antigens and shared neoantigen-reactive T-cell clonotypes with tumor-infiltrating TRM. Overall, the manuscript contains novel aspects, especially the recognition of neoantigen by the non-cancerous derived TRM, which is supported by strong data.

We thank the reviewer for the positive comments.

The difference of transcript expression and signature between TRM from FT and tumors is interesting, but not so impactful given the fact that several studies described the exhausted signature in tumor-infiltrating TRM from different cancer types. The TCR shared between T cells from FT and ovarian tumors is well demonstrated, but the description of results is sometimes confusing and lacks some comparison to fully support the conclusion. The link between the FT TRM and the protection and/or the prevention of cancer is not supported by solid data and is limited to the observation of TRM in the FT from four patients.

We thank the reviewer for raising this important point, but please consider that while the observation of exhaustion is well documented in other cancers, our observation is different as we do not simply report that cancer TILs are exhausted. We report that T cell clonotypes exhibiting exhaustion in tumors are also found in the tissue of origin, the fallopian tubes, where they display a markedly less exhausted phenotype. To our knowledge, this observation has not been reported in ovarian cancer or, more broadly, across solid tumors.
We acknowledge the limited patient population in this study (n = 4). We now discuss this in the manuscript as a limitation and note that, although small, the use of paired FT tumor samples with matched scRNA-seq and scTCR-seq allowed us to perform within-patient comparative analyses and clonotype-level evaluations (lines 619-630).

Major Comments

1. *Figure 1: It is surprising that any CD4 T regulatory cluster is found at least in the tumor compartment. The author should discuss or explain the absence of Tregs?*

Thank you for highlighting this point and we appreciate the observation that CD103 can be expressed by a subset of FOXP3$^+$ regulatory T cells. In our single-cell dataset, however, we do not identify a transcriptionally distinct Treg cluster, including within the tumor compartment. We believe this reflects both the targeted enrichment strategy and the biological context of the samples analysed. Our workflow selectively enriched for CD3$^+$CD103$^+$ tissue-resident memory–like T cells, within which FOXP3$^+$ Tregs are expected to represent only a minor and

heterogeneous fraction, potentially insufficient to resolve as a discrete cluster. In addition, FOXP3 expression in chronically inflamed tumor microenvironments has been reported to be unstable[1-3], which may result in dispersed FOXP3$^+$ cells rather than a cohesive Treg population. Consistent with this interpretation, our further analysis indicated that FOXP3 transcripts are detected at low levels in a limited number of individual cells and do not define a coherent cluster (Rebuttal Figure 1).

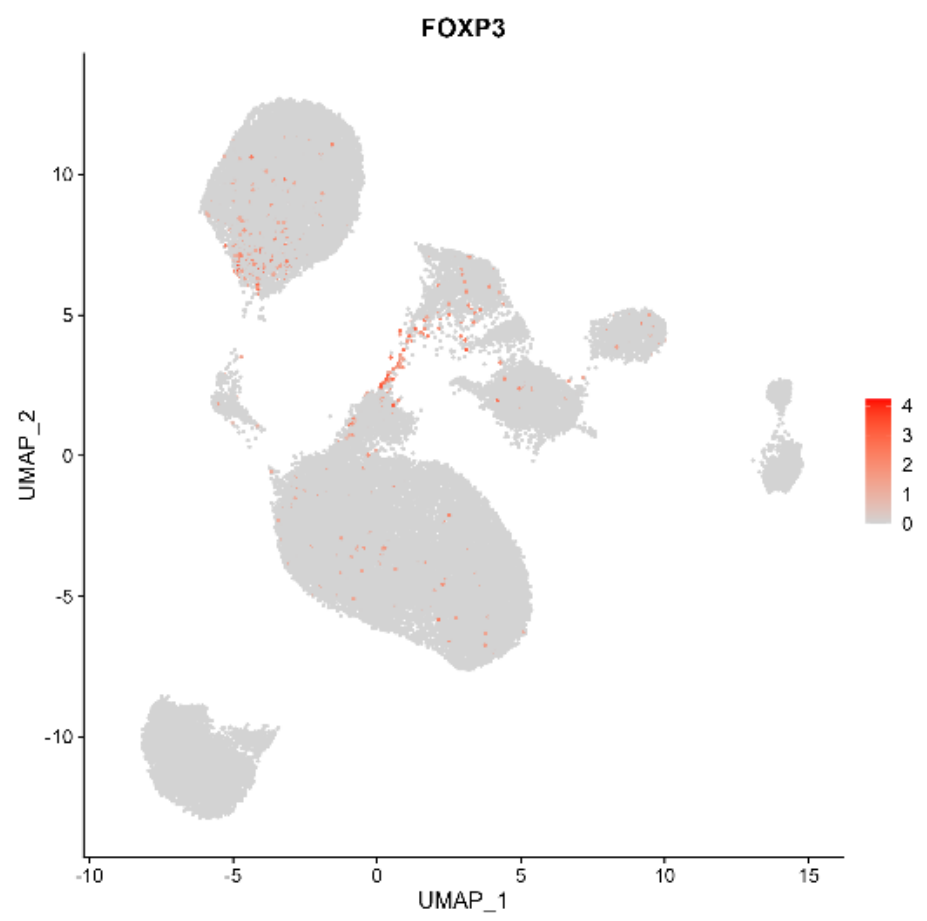

Rebuttal Figure 1. FOXP3 Feature Plot showing sparse FOXP3$^+$ cells and absence of a distinct Treg cluster.

2. The link in the figure between TRM and the overall scRNAseq is not clear. Do the scRNAseq were performed on total T cells from tissues and tumors or only on TRM? Which cell surface markers were used for the sorting of resident T cells? Authors should clarify this technical aspect as there is not TRM annotations on the scRNAseq analysis.

Thank you for pointing this out. In our study, we enriched for CD3$^+$CD103$^+$ tissue-resident memory-like (TRM-like) cells in both fallopian-tube (FT) and omental metastases, but profiled all CD3$^+$ cells in peripheral blood (PBMCs) due to the very low frequency of CD103$^+$ T cells in circulation. (Results, lines 112-117) Because TRM identity was determined by surface phenotype rather than by a single transcriptomic program, TRMLs do not form a discrete 'TRM cluster' but instead appear across multiple transcriptionally defined clusters. We have clarified this point explicitly in the revised Results:

1- (Lines 112-113: To enrich for tissue resident memory cells, we flow-sorted CD3+CD103+ cells from cancer and normal tissues.)
2- (lines 128-134: Although the FT and omental samples were pre-enriched for CD3$^+$CD103$^+$ TRMLs, TRMLs do not appear as a single transcriptionally defined TRM cluster. Instead, because TRM identity was determined by the sorting phenotype rather than by a unified transcriptomic program, TRMLs distribute across multiple functional clusters. For this reason, we annotated clusters according to their dominant transcriptional states (e.g., Tex, Tem, proliferating, MAIT, CD4-Th1) rather than labeling them as 'TRM'. Accordingly, clusters such as C1_CD8-Tex, C2_CD8-Tem, C4_CD8-SIK3, C5_CD8-CFD, C6_CD8-Prolif, C7_MAIT, and C10_CD4-Th1 represent TRMLs subsets derived from the CD103-enriched tissue samples.)

3- (Figure 1A legend: lines 1276-1277: Single cell suspensions from tissue were flow-sorted using CD3 and CD103.)

3. It is surprising that there is no detection of TRM clusters or Trm signature in the scRNAseq analysis and no description of Trm clusters was included in the main text. The authors should clarify in the result section which Trm clusters or T-cell clusters are described.

We appreciate this point. Because the FT and omental scRNA-seq datasets were derived from sorted $CD3^+CD103^+$ TRMLs, these cells appear across several transcriptionally defined clusters rather than forming a single TRM-labeled cluster. As detailed in the revised Results (lines 128–134), TRMLs are distributed among functional subsets such as C1_CD8-Tex, C2_CD8-Tem, C4_CD8-SIK3, C5_CD8-CFD, C6_CD8-Prolif, C7_MAIT, and C10_CD4-Th1.

4. Do the authors consider the MAIT cluster as TRM cells?

Yes—within our dataset the MAIT cluster represents tissue-resident MAIT (MAIT-TRM) rather than circulating MAIT. This is because: (i) we prospectively enriched for $CD3^+CD103^+$ T cells, (ii) the MAIT cluster expresses canonical MAIT markers (TRAV1-2, KLRB1/CD161, SLC4A10). We believe that this observation is of high interest in its own right.

5. Figure 2: If the scTCRseq was done only on TRM from tissues and T cells from PBMC, it makes difficult to conclude on the expansion comparison or TCR lineage as the T cells that are not TRM could may be also expand in the tumor tissue.

Thank you for this valuable comment. We agree that, because the FT and omental samples were enriched for $CD3^+CD103^+$ TRM-like cells whereas PBMCs were profiled as bulk $CD3^+$ T cells, clonal expansion levels should not be interpreted as a direct quantitative comparison across compartments. Our intention was to describe the expansion states within each compartment and to show where shared clonotypes reside rather than to compare absolute expansion between PBMC and tumor TRMLs.
To address this point, we have revised the Results to explicitly state this limitation and to clarify how expansion values should be interpreted (lines 229-237: Because FT and omental samples were enriched for $CD3^+CD103^+$ TRMLs whereas PBMCs represent total $CD3^+$ T cells, expansion levels are interpreted within each compartment rather than compared directly across compartments.)
This clarification does not alter our conclusions that hyperexpanded shared clonotypes are found only in tumors and map predominantly to TRML subsets such as C1_CD8-Tex, C4_CD8-SIK3, and C6_CD8-Prolif.

6. The tumor-reactive T cells argument is based only on the sharing TCR is not strongly convincing.

We agree that TCR sharing alone is supportive but not sufficient to define tumor-reactive T

cells. To strengthen this inference, we incorporated two orthogonal functional approaches and mapped reactive scTCR clonotypes to our datasets.

1.Autologous co-culture assay (ex vivo functional readout).

Sorted FT TRMLs ($CD3^{+}CD103^{+}$) were co-cultured with autologous tumor organoids. Upon tumor encounter, we observed antigen-dependent activation, including upregulation of CD137 (4-1BB) and induction of IFN-γ, compared with media controls and co-culture with non-malignant FT organoids. These results demonstrate the presence of tumor-responsive T cells within the FT TRML population.

2.Stimulation using tumor-specific mutations

To provide direct functional evidence of tumor reactivity, we performed whole genome sequencing and RNA sequencing of the tumor and mutation detection and expression analysis. We then made mutant and wild type peptide pairs of the detected neoantigens. We then tested whether these mutants stimulated TRMLs from the fallopian tubes in culture. We provide evidence of upregulation of CD137 (4-1BB) and induction of IFN-γ upon direct stimulation with tumor mutant peptides.

3.Reactive-cell scTCR mapping.

Following stimulation with autologous tumor organoids (as in 1) or predicted neoantigen peptides (as in 2), $CD137^{+}$ T cells were isolated and profiled by scTCR-seq. We found that the resulting 'reactive clonotypes' were enriched and shared among FT TRMLs and tumor TRMLs that were found in our original analysis of tumor-normal pairs. This provides a direct link between functional tumor reactivity and the T cells with shared TCRs across compartments.

Together, these functional and TCR-based analyses provide strong evidence that the T cells with shared FT-tumor TCRs include tumor-reactive clonotypes, going beyond inference based solely on repertoire overlap.

7. If TCR clonotype is shared between clusters, this means that tumor-specific T cells are distributed in all populations?

Not necessarily. While the TCR clonotypes are shared between clusters, this does not imply that tumor-specific T cells are present in every population. The tissue datasets consist primarily of TRM-like cells, and the clusters represent different functional or activation states within this TRMLs compartment. Thus, a single clonotype can appear in multiple states (e.g., effector-memory, proliferating, or exhaustion-skewed), reflecting expected state plasticity within TRMLs populations.

Importantly, we do not infer tumor specificity from TCR sharing alone. Instead, the suggestion that shared FT and tumor clonotypes may be tumor-reactive is supported by our functional assays and reactive-cell scTCR mapping, whereas sharing by itself reflects lineage relationships rather than definitive specificity.

8. The reading and conclusion of the paragraph describing figure 2 is not easy to follow and the overall message is difficult to catch.

We agree that the Figure 2 section contains several analyses and that the overarching message could be made clearer. To improve readability without altering the scientific content, we added

a brief introductory sentence to orient the reader to the overarching goal of the analysis—namely, assessing the degree of TCR overlap between FT TRMLs and tumor-infiltrating T cells (lines 184–186: To determine whether TRMLs in non-cancerous FT share lineage relationships with tumor-infiltrating TRMLs, we examined the extent, distribution, and functional characteristics of shared TCR clonotypes across FT, tumor, and PBMC.). We also added a concise summary sentence that highlights the key conclusion of the section: the preferential and functionally relevant overlap between FT and tumor TCRs, compared with PBMC(lines 254-255: Together, these analyses demonstrate that FT TRMLs show preferential and functionally relevant TCR overlap with tumor-infiltrating T cells, distinct from PBMC sharing patterns.). These minor clarifications improve the flow of the section and make the main conclusion easier to follow.

9. Figure 3: which gene or features distinguish the two cluster of T-ex? What is the rational to split this cluster in two clusters?

As shown in Figure 3D, the two exhausted TRMLs subsets differ in their expression of key exhaustion-associated genes. In particular, Tex2 shows higher expression of CXCL13, ZNF704, CTLA4, and HAVCR2 (TIM-3) compared with Tex1.
We separated the exhausted TRMLs compartment into two states because trajectory analysis revealed a progression within the Tex population, with Tex2 representing a more advanced exhaustion state. This is supported by differences in marker genes, exhaustion-related gene-set scores, and pseudotime positioning, all of which indicate that Tex1 and Tex2 reflect early versus more terminally exhausted TRM states rather than a single homogeneous cluster. We have added a sentence to the Results to clarify this rationale (lines 261-263: Because trajectory analysis revealed internal heterogeneity within the exhausted TRMLs population, we resolved the original CD8_Tex cluster into two transcriptionally distinct states, CD8_Tex1 and CD8_Tex2.)

10. Figure 3G: Is the proportion of CD8 SIK3 positive representative of the cluster proportion in the scRNAseq? It would be interesting to validate the phenotype switch of this CD8 T-cell population at the protein levels and/or with in vitro experiments.

In Figure 3G, the immunofluorescence images demonstrate protein-level expression and subcellular localization of SIK3 in $CD8^+$ T cells, but do not quantify population-level frequencies due to the limited number of cells per field. To address proportional abundance directly, we performed additional flow cytometry analyses across tissues and patients. These data show that 46–98% of $CD8^+$ T cells express SIK3 at the protein level (depending on patient and tissue; Figures S4-S5), consistent with the substantial $SIK3^+$ proportion observed in the corresponding scRNA-seq cluster.

We also validated the phenotypic transitions inferred from scRNA-seq using in vitro differentiation assays in primary patient-derived $CD8^+$ T cells. Under TRM-supportive stimulation (TGF-β + IL-15)[4], $CD8^+$ T cells reproducibly progressed from a TRM-like state to Tex1, then into a $SIK3^+/TOX^+$ intermediate state, and finally toward a terminal Tex2 phenotype

(Figures S4-S5), concurring with the trajectory observed in pseudotime (Figure 3A-D). These results confirm that the SIK3-high state represents a physiologically inducible intermediate exhaustion phenotype at the protein level. We have incorporated a concise summary of these findings into the Results (lines 336–347:To further validate the phenotypic transitions inferred from scRNA-seq, we performed protein-level in vitro differentiation assays using $CD8^+$ T cells from FT, ovary, and omentum of two HGSOC patients under TGF-β + IL-15 stimulation. Across tissues, $CD8^+$ T cells followed a conserved exhaustion trajectory at the protein level that aligned with the scRNA-seq trajectory analysis (TRM to CD8_SIK3/Tex2), with tissue-specific differences in magnitude (Figures S4, S5). FT displayed TRMLs to Tex1 to CD8_SIK3 progression, whereas ovary showed a proliferative exhaustion pattern with coordinated T-SIK3 and Tex2 induction. Omentum exhibited the strongest de novo Tex2 exhaustion, marked by high expression and fold-change of TIM3, PD-1, CXCL13, and CTLA4. Ki67 expression levels changed minimally, consistent with cells already positioned along exhaustion trajectories. Although both patients followed the same trajectory, patient 11699 showed stronger T-SIK3/TOX induction, whereas patient 11705 demonstrated a more pronounced terminal Tex2 phenotype. These findings confirm that the SIK3-high state is a reproducible intermediate exhaustion phenotype at the protein level.).

11. Figure 3H: The conclusion on the clonal diversity within the cluster CD8_SIK3 is not clear. What is the main message?

The main message of Figure 3H is that the CD8_SIK3 cluster contains substantially greater clonal diversity in tumors than in FT. After depth normalization, the tumor-derived CD8_SIK3 subset harbors a larger number of unique (non-shared, private) clonotypes, whereas the FT-derived CD8_SIK3 subset contains fewer distinct clonotypes. This indicates greater clonotype richness and heterogeneity within the tumor SIK3 population. This difference may reflect greater antigenic diversity or selective pressures within the tumor microenvironment, although additional studies will be required to define the underlying mechanisms.

12. Because this cluster is at the end of the pseudotime trajectory, what is the hypothesis about the fact that these cells are not expanded?

We did not conclude that the CD8_SIK3 cluster, despite being positioned at the end of the pseudotime trajectory, is ‘not expanded’. Pseudotime reflects transcriptional progression, not clonal expansion, and therefore cannot be used to infer clone size. Figure 3H shows clonal diversity within the CD8_SIK3 cluster, but this does not imply a lack of expansion. To directly assess expansion, we examined TCR clonotype frequencies. As shown in Figure 2J and in the newly added clonal occupancy analyses provided in Figure S3I-J, expanded and hyperexpanded shared clonotypes are indeed present within the tumor-derived CD8_SIK3 population. Thus, the terminal pseudotime position of CD8_SIK3 reflects a distinct transcriptional fate rather than an absence of clonal expansion.

13.Why the percentage of unique clone is even higher in the tumor tissue while these T cells undergo more differentiation and exhaustion switch? How is the heterogeneity on the percent of the cluster and TCR clonality between patients?

The percentage of unique clones and the degree of differentiation/exhaustion reflect different biological properties. Unique clone richness measures the number of distinct clonotypes detected at matched depth, whereas exhaustion is a transcriptional state and clonal expansion is quantified by frequency-weighted TCR metrics. These properties are not mutually exclusive: a tissue can contain many low-frequency unique clones while a smaller subset of expanded clones drives the exhausted phenotypes.[5,6]

The higher proportion of unique clonotypes in tumors likely reflects the broader and more dynamic antigenic landscape of the tumor microenvironment. Tumors continually generate and reveal new antigenic determinants through neoantigen evolution and epitope spreading[7], which can recruit additional T-cell clonotypes that often remain at low frequency. Moreover, inflamed tumors attract diverse bystander or weakly tumor-reactive T cells[8], further increasing repertoire richness without necessarily contributing to exhaustion or clonal dominance.

Regarding inter-patient heterogeneity, differences in cluster composition and clonotype distributions across patients are shown in Figures S2D-F and S3B. These data illustrate patient-specific variation in both T-cell subset proportions and clone-size distributions, which is expected given individualized immune histories and tumor microenvironments.

14.Authors claim that CD8_SIK3 cluster and more precisely the shared TCR clonotypes undergo a specific upregulation of some genes depending of the environment. What is the effect of this environment on others cluster?

We focused on the CD8_SIK3 subset because, unlike the well-characterized T_ex and T_em clusters, this TRML subset population has not been previously described, and we therefore examined it in greater depth. In doing so, we observed that CD8_SIK3 cells exhibited a clear transcriptional trajectory and environment-associated gene modulation based on our pseudotime and differential expression analyses. We did not perform equivalent environment-specific differential expression analyses for all other clusters, nor did we pursue systematic gene-module characterization across subsets, as our primary goal was to highlight the unique features of the CD8_SIK3 population. Further investigation of how different TRML subsets respond to the FT and tumor microenvironments will be an important direction for future work.

15. Is T_ex also have a similar activation trend in the tumor compared to FT?

Based on our existing analysis (Figure 4F), exhaustion and activation markers are generally upregulated in tumor-derived shared TRMLs compared with FT. Given that the T_ex subset represents the largest proportion of shared TCR-bearing cells, it is likely that T_ex cells follow a similar activation trend in the tumor microenvironment. A more detailed, subset-specific differential expression or GO analysis of T_ex cells and other TRML subsets would be valuable

and represents an important direction for future work.

16. What is the effect on non-shared TCR clonotypes? Especially given the fact that most of the TCR of these clusters are unique.

Our analysis focused on shared TCR clonotypes because they provide a unique opportunity to directly compare the same antigen specific T cell clones across FT and tumor, allowing us to isolate environment-associated transcriptional differences. In contrast, non-shared clonotypes are not paired between FT and tumor and represent a diverse set of TCR specificities. Because these clones likely recognize different antigens and exhibit substantial transcriptional heterogeneity, combining them into a single group for differential expression or pathway analysis would not yield a biologically interpretable comparison of microenvironmental effects. While non-shared clonotypes consistently showed lower activation and exhaustion signatures relative to shared clonotypes in our existing analyses, a detailed investigation of their environment-specific transcriptional programs would require a study design specifically tailored to antigen-specific heterogeneity and is an important direction for future work.

17.In the same line, the authors should demonstrate that the exhausted and activation features upregulated in tumors, and thus the tumor-reactive signature, is not happening in the non-shared TCR cells (FT vs tumor) to argue that the tumor-reactivity is restricted to shared TCR-expressing cells.

We agree that demonstrating that the tumor-reactive transcriptional program is specific to shared TCR clonotypes is desirable. Our existing analyses already address this directly. In Figure 4B-C, shared clonotypes display markedly higher expression of exhaustion and activation markers (including CXCL13, PDCD1, CTLA4, TIGIT, HAVCR2, and TOX) compared with non-shared clonotypes across all TRMLs. In Figure 4F, we further show that these tumor-associated features are selectively upregulated in shared clonotypes in the tumor relative to FT.

Importantly, non-shared clonotypes do not exhibit the same tumor-reactive profile. Supplementary Figure S4A-C shows that in tumors, the exhaustion/activation signature is strongly enriched in shared clonotypes, whereas non-shared clonotypes maintain substantially lower expression of these markers. Similarly, in FT and PBMC, shared clonotypes consistently show higher activation/exhaustion features than non-shared TCRs.

Together, these results demonstrate that the tumor-reactive transcriptional program is preferentially associated with shared TCR clonotypes and is not broadly induced in non-shared TCR cells, supporting the interpretation that shared FT and tumor clonotypes may represent the tumor-responsive T cell population.

18. Authors discussed the possibility that SIK3-expressing TRM cells exhibit a transition state of differentiation before exhausted state, however the SIK3-TRM appear at the end of the trajectory analysis, which does not support this hypothesis.

In our analysis, SIK3-high TRMLs do map to a high pseudotime position; however, they do

not exhibit the transcriptional profile of terminally exhausted cells. Instead, they represent a late, but non-terminal, exhaustion-associated state that is transcriptionally distinct from the canonical terminal exhaustion program. This resolves the apparent discrepancy:

Pseudotime provides a continuous ordering based on progressive transcriptional change relative to a defined root, whereas our exhaustion phase annotations reflect discrete transcriptional states.

Thus, a cluster may localize near the end of pseudotime while still being separable from terminal exhaustion. In our dataset, SIK3-high TRMLs display epigenetic- and metabolic-remodeling signatures consistent with a late transitional, rather than terminal, exhaustion state. We have clarified this interpretation in the revised text (lines 580-582: Although SIK3-high TRMLs map toward the end of the pseudotime trajectory, their transcriptional profile lacks the defining features of terminal exhaustion, consistent with a late transitional rather than terminal state.).

19. Moreover, this cluster seems more present in the tumor tissue compared to FT, in an opposite trajectory compared to T-ex2. Authors should clarify the relationship between SIK3-expressing and T_ex clusters. How is the TCR sharing between these clusters and how it changes in the FT or the tumor?

We have added a summary table of shared TCRs cluster-wise cell counts across samples (FT, PBMC, Tumor; Table S3) and report the distribution of shared clonotype cells across clusters (Figure 2E,J; Figure S3I–J). Consistent with the reviewer's observation, the CD8_SIK3 cluster is more abundant in tumor than FT (227 vs. 59 cells), while the C1_CD8-Tex cluster shows strong enrichment in tumor-shared clonotypes (4,631 tumor cells; 1,410 FT cells). To clarify the relationship between these states, we quantified TCR overlap between them and identified 66 clonotypes present in both CD8_SIK3 and CD8_Tex. These overlapping clonotypes are more frequently represented in tumor samples, suggesting that SIK3-expressing and Tex cells share common clonal origins but diverge into distinct transcriptional states in response to the tumor microenvironment.

In addition, shared clonotypes were also observed in other TRML subsets, including CD8_Tem, which is consistent with the inferred Tem to Tex1 to Tex2/SIK3 branching trajectory. This distribution reflects the expected progression of individual clones through multiple intermediate states rather than discrete, unrelated populations. A summary of these clonotypes is provided in Table S4. We have added this interpretation in the revised text (lines 312-317).

20.It would be interesting to estimate the level of neoantigens present in FT and in tumors to explore the impact of antigen expression and chronicity on the differentiation state of TRM.

Thank you for this insightful suggestion. Following your recommendation, we quantified predicted neoantigen levels in both FT and tumor samples from patients 11642 and 11657. Across both patients, the total number of predicted neoantigens was comparable between FT and tumor; however, the prevalence of these neoantigens was substantially lower in FT. As the prevalence threshold increased, neoantigen load in FT rapidly declined to zero, whereas tumor samples retained neoantigens even at high prevalence thresholds. When using a prevalence

cutoff ≥0.3, the tumor consistently exhibited markedly higher neoantigen load compared with FT.

These findings are consistent with the more exhausted phenotype observed in tumor-resident TRMLs, potentially reflecting chronic exposure to higher-prevalence antigens in the tumor microenvironment. However, we cannot make a direct mechanistic link between predicted antigen expression/chronicity and TRM differentiation, as our predictions do not determine whether the low-prevalence neoantigens are truly immunogenic. We have incorporated this analysis and clarification in the revised manuscript (lines 464-474: To further investigate the relationship between genomic alterations and potential antigenic drivers of TRMLs differentiation, we next quantified predicted neoantigen load in both FT and tumor samples. Although the overall number of predicted neoantigens was similar between FT and tumor, their prevalence differed markedly. Across both patients, neoantigen load in FT rapidly decreased to zero as prevalence thresholds increased, whereas tumor samples retained neoantigens even at high prevalence levels. At prevalence more than 0.3, tumors harbored substantially more predicted neoantigens than FT(Figure S9 E). These results suggest that while FT contains a broad set of low-prevalence mutations, the tumor microenvironment is characterized by a higher burden of prevalent neoantigens, consistent with the more exhausted phenotype observed in tumor-resident TRMLs. However, because the immunogenicity of these predicted neoantigens cannot be confirmed computationally, we cannot directly infer the impact of predicted antigen expression or chronicity on TRM differentiation.).

Minor:

21. Figure 1: There is a problem of figure panel correspondence in the legend. Some legends in the figure are too small leading to difficulty to understand the information. Lack of title or legend on supplementary tables.

We have corrected the panel legend correspondence in Figure 1 and improved the readability of all figure labels by increasing font size where needed. In addition, we have added clear titles and legends to all supplementary tables to ensure the information is easy to interpret.

Reviewer #2 (Remarks to the Author):

I co-reviewed this manuscript with one of the reviewers who provided the listed reports. This is part of the Nature Communications initiative to facilitate training in peer review and to provide appropriate recognition for Early Career Researchers who co-review manuscripts.

Reviewer #3 (Remarks to the Author):

This carefully constructed manuscript takes a deep dive into T cell dynamics in high-grade serous ovarian cancer (HGSC). This study is addressing a clinically relevant question - despite remarkable genomic instability, these tumors are generally 'immune cold' and don't respond well to PD1/PDL1 checkpoint blockade but we have a poor understanding of the T cell dynamics during the evolution of these tumors. Although this report used a relatively small sample size for initial discovery (4 patients), the team profile multiple tissues across the cohort using single cell sequencing of transcriptomes and TCRs to map clonality and differentiation of T cells in likely tissue of origin (FT), tumors and omental metastases. A number of key

findings are reported including a novel SIK3+ T cell state that may represent a distinct exhaustion trajectory in HGSC, and a high degree of sharedness between fallopian T cell TCRs and tumor TCRs reflecting tumor origins. The study is thorough, using orthogonal assays to validate/extend key findings. The new SIK3+ subset is validated by IF and the team also use sophisticated autologous co-culture studies to validate key findings and identify potential neoantigen targets of expanded TCR clones. Overall, this is an important and non-incremental study that sheds light on T cell dynamics in HGSC and could lead to the development of new immunomodulatory therapeutic strategies for these highly lethal tumors. There is great enthusiasm for this report, with a few opportunities for improvement through minor revision, as follows –

1. On the whole the statistical analyses are thorough but in two places the team could consider using more rigorous methods
a. Paragraph starting on 138 – there are tools to compare cell composition and subset abundance (MILO, Speckle and others) that take interpatient variability into account

Thank you for this insightful suggestion. In response, we incorporated Speckle/Propeller analysis to compare cell composition and subset abundance while accounting for inter-patient variability (Table S1). We updated the manuscript accordingly. Using propeller_ttest, we found no statistically significant differences in the abundance of individual clusters between FT and tumor samples, indicating broadly similar TRML compositions across these sites. However, propeller_anova_by_tissue revealed that most clusters differed significantly across FT, tumor, and PBMC samples, with the exception of clusters C5, C6, C9, and C10. This was expected, as PBMCs were sorted as bulk $CD3^{+}$ T cells, whereas FT and tumor samples were enriched for $CD3^{+}CD103^{+}$ TRMLs. These results have now been incorporated into the revised text (lines 157-166) and Table S1.

b. Paragraph starting line 351 – the authors could consider testing if the overall composition of data points are different in FT versus HGSC, rather than contrasting correlations (it may be that the effects go in a similar direction for example, but with a smaller magnitude)

Our analysis in this section aimed to compare the transcriptional programs associated with shared TCRs to previously validated neoantigen-reactive signatures, rather than to test compositional differences between FT and tumor gene sets directly. To quantify these relationships, we used Gene Set Variation Analysis (GSVA) to compute single-cell gene set enrichment analysis (GSEA) scores for each gene set, and then assessed the similarity of these enrichment profiles by Pearson correlation, as described in the Methods (lines 849-850). This approach evaluates the overall transcriptional alignment between our FT and tumor derived signatures and the published NeoTCR and neoPBL reference signatures.

2. Figures:
a. Many of the figures have very small text, which was challenging to read when printed, for accessibility it is recommended to increase the font size throughout
We have improved the readability of all figure labels by increasing font size where needed.

b. On the UMAPs, including the n cells can help the reader to better help the reader contextualize the data and understand the impact of any filtering

We have now added the number of cells (n) to all UMAP visualizations to help contextualize the dataset and filtering steps.

c. Figure 5 panel H needs a more detailed figure legend for readers not familiar with this assay/data type

We have revised the figure legend as follows:

(H) Left: Deconvolution of peptide pool responses, showing the reactivity of individual peptides. Two of the 27 predicted neoantigen candidate peptides elicited detectable IFN-γ production from FT-derived TRMLs, indicating the presence of pre-existing neoantigen-specific T cells. Right: Quantification of IFN-γ ELISpot responses, presented as the number of spots per 2×10^5 TRMLs (mean ± SEM). Statistical significance was determined using a Student's t-test ($P < 0.008$).

3. A code availability statement was included but there are no details of data availability.

Although data availability information was included in the summary report, it was missing from the manuscript. We have now added the data availability description in the revised version (lines 660-663).

Reviewer #4 (Remarks to the Author):

The article by Wang et al. describes tissue resident memory T-cells (TRMLs) and their hypothesized role within HGSOC development. The authors show the presence of these TRMLs within (healthy) fallopian tube tissue of HGSOC patients (n=4), and indicate towards their tumor responsiveness and lower exhaustion profile in comparison to TRMLs within omental tumors. However, the impact of these results are limited due to some important considerations which are lacking. For example, the only immune cells that are focused on are CD3+ CD103+ T-cells, while many other immune cell populations have been found to play an important role in HGSOC. Moreover, the conclusions made in this article are still widely based on a lot of assumptions and hypotheses, and should be reevaluated. Overall, this study uncovers a potentially important immune cell population within early development of HGSOC but should be reported differently with more scientific facts and more limited assumptions. Specifically, the article presents these cells as a solution for immunotherapy in these patients, which is a big leap from the findings presented here in a limited patient population. The article should be written more fundamentally.

Some major comments:

1. The first assumption lies in the importance of these TRMLs. As STIC lesions are usually non-invasive in early ovarian tumor development, the authors hypothesize that these lesions are contained due to the presence of TRMLs.

We thank the reviewer for raising this important point. We agree that our data do not establish

causality between TRML presence and the containment of STIC lesions. In the revised manuscript, we clarified that TRMLs represent one candidate contributor to local immune surveillance and acknowledged that additional immune subsets (e.g., macrophages, dendritic cells, NK cells, B cells) may also play essential roles in early HGSOC immunity. Our intention is to describe TRMLs as a potentially relevant population rather than the sole determinant of STIC confinement, and we have adjusted the text to reflect this(lines 86-88,93-94).
Please also note that we present this hypothesis/assumption in the introduction to inform the reader of the motivation of carrying out the subsequent analyses. After performing the analyses, we do not conclude that TRMLs are the only cells that constrain STICs. We have now made this clearer throughout the manuscript.

2. While this may be correct, this should be placed in the wider context. In general, early stage cancer is known to induce an anti-cancer immune response which can often be evaded or escaped by tumor cells when disease progresses. HGSOC patients are often diagnosed at a late stage of the disease in which immune escape and immunosuppression are highly abundant. Herein, many non-lymphoid immune cells play an important role, which is left untouched in this study.

Thank you for pointing this out. Our study was intentionally focused on $CD3^{+}CD103^{+}$ tissue-resident memory T cells in the fallopian tube, given their established relevance in epithelial barrier immunity and cancer prevention. Please see the report that we cited Thomas Gebhardt group's research on how TRMs prevent melanoma formation.[9] We agree that early-stage cancers elicit broader anti-tumor immune responses and that multiple non-lymphoid populations—including macrophages, dendritic cells, NK cells, and stromal cells—play key roles in shaping immune escape and immunosuppression in advanced HGSOC. Our analyses and conclusions do not imply primacy or sufficiency of TRMLs; rather, we aim to characterize their potential contribution within this wider immune landscape. To address the reviewer's point, we have added text acknowledging the roles of additional immune subsets and adjusted wording to situate TRMLs within the broader context of early immune responses in HGSOC. (lines 86-88,93-94).

3. The TRMLs evaluated in this study are derived from HGSOC patients (during debulking surgery? In what disease stage?).

All patients underwent interval debulking surgery following 3–4 cycles of neoadjuvant chemotherapy (carboplatin/paclitaxel ± PLD) with high-grade serous ovarian cancer (HGSOC) diagnosed at FIGO stage IIIC or IVa. We have now included this clinical context in the manuscript for clarity (lines 667-673).

4.While they are derived from 'non-tumoral fallopian tubes', cancer cells are present within the body of these patients. It should be discussed that the similar TCR expression and tumor recognition by the FT TRMLs can be related to neoantigens/TAAs being present within the peritoneal cavity/ascites of these patients? And can therefore not be extrapolated to the situation in early stage HGSOC? The results don't directly indicate the anti-tumor effect of

these cells in early disease stages, as this is not what is being evaluated here. But this is one of the main conclusions of this paper and should be nuanced more! Moreover, again early stage HGSOC is not often diagnosed so relevance of this is lacking.

We thank the reviewer for raising this important point, but please note that extrapolation to early stage HGSOC was based on clonality analysis and subsequent functional analysis. Please consider that when characterizing tumor neoantigens, we conducted prevalence analysis and only selected mutations that were clonal (i.e. present in all tumor cells) and were, therefore, present at the stem of the evolutionary tree of tumor development. We clearly show that two of these "founder" mutations elicited strong IFN-γ and CD137 responses. Please see figure 5H. Furthermore, single cell TCR sequencing of these reactive clones was found both in the tumor and fallopian tube TRMLs. Please note that clonality analysis is an established method to infer tumor evolutionary trajectories.[6,10,11] We exploit these evolutionary methods to show immune reactivity to "founder" mutations.

It is indeed possible that FT TRMLs from these late-stage HGSOC patients have been exposed to tumor-derived neoantigens or TAAs present within the peritoneal cavity or ascites. At the same time, several observations argue that the FT TRML population is not simply the result of passive spillover or bystander activation. The shared clonotypes express strong tissue-resident programs (e.g., ITGAE/CD103, residency-associated transcriptional signatures) and show substantially greater overlap with tumor-infiltrating lymphocytes than with circulating T cells. These features suggest that the FT TRMLs represent bona fide resident populations that have undergone local antigen experience within the FT epithelium rather than being recruited from ascites or blood.
However, we now explicitly discuss this limitation and acknowledge this in the revised manuscript (lines 619-630).

5. I do agree on the potential importance of these FT TRMLs for adoptive T-cell transfer after surgery due to their lower exhaustion and tumor responsiveness. This is an interesting approach to further explore, but should be placed within the wider immunological environment of HGSOC as T-cells alone may not be enough to overcome the high immunosuppression.

We agree with the reviewer that FT-derived TRMLs, while promising due to their lower exhaustion and intrinsic tumor reactivity, would be unlikely to achieve meaningful therapeutic activity on their own within the profoundly immunosuppressive microenvironment characteristic of advanced HGSOC. To address this, we have revised the Discussion to situate FT TRML based strategies within the broader immunological landscape of HGSOC and to emphasize that any future adoptive T-cell approaches would need to be deployed alongside modalities that relieve myeloid-driven suppression and chronic inhibitory signaling. Our revised text now frames FT TRMLs as a potential component of combination immunotherapy rather than as a stand-alone solution(lines 539-547).

Comments:

- Introduction:

6. Would suggest to already indicate to the high immunosuppression in HGSOC TME in the introduction and more background on what is already known immunologically.

We thank the reviewer for their suggestion. We have now added text in the Introduction noting that the HGSOC tumor microenvironment is highly immunosuppressive and shaped by multiple immune and stromal populations. This addition provides a broader immunological context for why anti-tumor T-cell responses often fail in advanced disease (lines 59-63).

- M&M:

7. please give more information on the patient population used. The immune landscape can differ greatly in patients depending on their disease stage and previous treatment cycles.

More detailed clinical information for each patient is provided in the summary report, and we have now added additional information on the patient cohort, including disease stage, treatment history, and type of surgery in the Methods section (lines 667-673).

8. For the FT, how did you confirm that the biopsy used in your study did not contain tumors? IHC? Can this be added in the supplementals? This is only described in the results rection, please provide details in M&M. Moreover, did you consider comparison of immune landscape in STIC lesions as well?

All samples were obtained from clinicians, and clinical pathologists confirmed the patient stage and that the FT tissues used in this study were non-tumoral based on routine diagnostic evaluation using the well established Sectioning and Extensively Examining the Fimbrial ends of the Tubes (SEE FIM). No invasive carcinoma or STICs were found by SEE FIM examination. The viable fallopian tube tissue biopsy was used for making FT organoids. These organoids were examined using whole genome sequencing that confirmed the absence of somatic mutations. The results were reported in the manuscript. Importantly, the absence of histologically detectable STIC lesions does not preclude prior antigen exposure, as immune recognition and memory formation may occur in response to early or transient pre-neoplastic events before morphologically identifiable lesions arise.

We agree that comparative analysis of immune landscapes in STIC lesions would be highly informative; however, no STICs were present in this cohort, precluding such analyses. Incorporating spatial transcriptomics or targeted approaches to study STIC-associated immunity represents an important future direction.

9. It seems like a bit of a stretch to assume that the TRMLs that you find in healthy fallopian tube tissue (but derived from cancer patient) are the same cells that contain the invasiveness of STIC lesions without actually evaluating these STIC lesions themselves? Same goes for the TRMLs at early disease stages. How can you be sure that these are similar to the TRMLs that you analyze here which are derived from advanced stage patients?

As mentioned previously in response to major concern number 1, we do not make a claim that the T cells in the FT constrain STICs. We simply used this as a motivation for the study.

It is impossible to know what state the T cells were in at transformation. However, as mentioned in response to point number 4, our tumor evolution analysis combined with functional work on TRMLs demonstrates that TRMLs exhibit significant response to mutations that occurred at the trunk (mutational prevalence = 1, Figure 5C-D,G-H) of the tumor evolutionary tree. This provides strong support for the notion that early transformation is recognized by adaptive immune responses. To our knowledge, we are not aware that this finding was previously established. Future work will focus on whether TRMLs constrain STICs and/or other preneoplastic states.

10. Please add the amount of patients and amount of samples used per assay in the M&M and results.
We have now added the number of patients and the amount of samples used for each assay in both the Methods and Results sections (lines 667–673).

11. scRNAseq was only performed on HGSOC tissue? So only on tumor tissue and not FT? be more specific in description of the materials used.
We have now clarified in the Methods that scRNA-seq and scTCR-seq were performed on FT, omental tumor, and peripheral blood samples from the same HGSOC patients (lines 667–673).

12. Why did you opt to take tumor-free FT and omental tumors? Why not directly compare tumor-free and tumor-bearing fallopian tubes? Can the fact that the tissue is different not explain why there are many more TRMLs in the tumor-free FT compared to the omental tumor? As the omentum has much less epithelial area for the TRMLs to adhere to?

We agree that the FT and omentum differ in epithelial density and microanatomy, which likely contributes to the higher proportion of $CD103^{+}$ TRMLs in the FT. We do not infer any functional consequences from these differences. We have now noted this in the manuscript and clarified that tissue architecture may influence TRML abundance (lines 121–123).

13. Line 138, there is no Figure 1F?
Figure 1 was mislabeled, and we have corrected it in the revised manuscript.

14. Quality of the figures should be improved. Some legends are not readable.
We have improved the overall figure quality and increased the font size of labels and legends to ensure readability in both print and digital formats.

- Discussion
15. It is important to discuss the importance of your findings. For example, results from figure 3, while impressively gathered and interesting, are not very surprising. The fact that exhaustion of immune cells is higher in the tumor region compared to the non-tumoral area seems quite logical, as the non-tumoral area is exposed less to inflammation, cytokines and antigens release.

We agree that increased T-cell exhaustion in tumor tissue compared to non-tumoral FT is expected, given the greater antigen load and inflammatory cues present in tumor sites. However, we respectfully disagree that our finding is “not very surprising”. The difference in the exhaustion state in its own merit may not be surprising but the context is critical. There is no obvious or previously documented reason to believe that one would find tumor reactive clones in the non-cancerous fallopian tubes that are identical to those found in the vicinity of the tumor. Furthermore, the contribution of our analysis is to quantify these differences within TCR-defined clonotypes that are shared between FT and tumor, demonstrating that the same antigen-experienced clones display markedly different exhaustion states depending on tissue context. This paired comparison is not captured by bulk or cluster-level analyses alone and reveals that FT TRMLs preserve a less exhausted, more responsive phenotype despite sharing TCRs with tumor-infiltrating TRMLs. We have added clarifying text to the Discussion to emphasize this conceptual contribution (lines 532-537).

16. Please discuss if there is any indication that these TRMLs are also this abundantly present in earlier stages in the disease, proven in other paper? You have only shown advanced disease.

Direct evidence on TRM or TRML abundance in early-stage fallopian tube tissue is limited. A recent multimodal spatial-profiling study of FT precursor lesions (p53 signatures and STIC) reported immune activation and early immune surveillance features[12], but did not specifically characterize CD103^{+} TRM/TRML populations or tumor specific responses.

17. Please discuss limited patient population.

We acknowledge the limited patient population in this study (n = 4). We now discuss this in the manuscript as a limitation and note that, although small, the use of paired FT tumor samples with matched scRNA-seq and scTCR-seq allowed us to perform within-patient comparative analyses and clonotype-level evaluations (lines 619-630).

18. When discussing the potential of these TRMLs for use in vaccinations or preventative therapy, it is important to discuss the relevance of this in HGSOC, which is very rarely found at a stage that preventative measures can still be used. Focus could be more on the adaptive T-cell transfer therapy as this is more logical and supported more by your results.

In the revised Discussion, we clarify that preventive vaccination is proposed as a conceptual strategy for high-risk individuals (e.g., BRCA mutation carriers or those undergoing risk-reducing surgery), rather than as an intervention for clinically detected early-stage HGSOC, which is indeed rare. Our intention is to highlight how the preserved functionality and tumor reactivity of FT-derived TRMLs provide a biological rationale for priming naïve or resident T cells against neoantigens or TAAs prior to malignant transformation. This concept aligns with emerging efforts to develop neoantigen- and TAA-based preventive vaccines[13], including ongoing work in our laboratory. Consistent with the reviewer’s suggestion, we have refocused the translational discussion on adoptive transfer of FT-derived TRMLs as the more immediate

and data-supported application, while presenting preventive vaccination as a longer-term conceptual extension.(lines 537-547, 560-563).

19. It is indicated in the reporting summary that all patients received NACT, meaning they have received multiple cycles of chemotherapy before debulking surgery in which the samples were taken? Please disclose how the chemotherapy regimen can impact the immune cells.

It is correct that all patients underwent neoadjuvant chemotherapy (NACT) prior to interval debulking, and all samples were collected at the time of surgery. We acknowledge that NACT can influence antitumor immunity through mechanisms such as transient lymphodepletion, reshaping of myeloid populations, and induction of immunogenic cell death. Because our study design relied on paired sampling within the same patients, the major comparative analyses (FT vs. tumor; shared vs. non-shared TCRs) remain internally controlled. Nevertheless, we now explicitly note in the manuscript that prior chemotherapy may modulate the immune landscape and represents an important limitation to consider in interpreting our findings (lines 619-630).

Reference:

1. Beres, A., Komorowski, R., Mihara, M., and Drobyski, W.R. (2011). Instability of Foxp3 expression limits the ability of induced regulatory T cells to mitigate graft versus host disease. Clin Cancer Res *17*, 3969-3983. 10.1158/1078-0432.CCR-10-3347.
2. Zhou, X., Bailey-Bucktrout, S.L., Jeker, L.T., Penaranda, C., Martinez-Llordella, M., Ashby, M., Nakayama, M., Rosenthal, W., and Bluestone, J.A. (2009). Instability of the transcription factor Foxp3 leads to the generation of pathogenic memory T cells in vivo. Nat Immunol *10*, 1000-1007. 10.1038/ni.1774.
3. Downs-Canner, S., Berkey, S., Delgoffe, G.M., Edwards, R.P., Curiel, T., Odunsi, K., Bartlett, D.L., and Obermajer, N. (2017). Suppressive IL-17A(+)Foxp3(+) and ex-Th17 IL-17A(neg)Foxp3(+) T(reg) cells are a source of tumour-associated T(reg) cells. Nat Commun *8*, 14649. 10.1038/ncomms14649.
4. Mackay, L.K., Wynne-Jones, E., Freestone, D., Pellicci, D.G., Mielke, L.A., Newman, D.M., Braun, A., Masson, F., Kallies, A., Belz, G.T., and Carbone, F.R. (2015). T-box Transcription Factors Combine with the Cytokines TGF-beta and IL-15 to Control Tissue-Resident Memory T Cell Fate. Immunity *43*, 1101-1111. 10.1016/j.immuni.2015.11.008.
5. Sade-Feldman, M., Yizhak, K., Bjorgaard, S.L., Ray, J.P., de Boer, C.G., Jenkins, R.W., Lieb, D.J., Chen, J.H., Frederick, D.T., Barzily-Rokni, M., et al. (2018). Defining T Cell States Associated with Response to Checkpoint Immunotherapy in Melanoma. Cell *175*, 998-1013 e1020. 10.1016/j.cell.2018.10.038.
6. Yost, K.E., Satpathy, A.T., Wells, D.K., Qi, Y., Wang, C., Kageyama, R., McNamara, K.L., Granja, J.M., Sarin, K.Y., Brown, R.A., et al. (2019). Clonal replacement of tumor-specific T cells following PD-1 blockade. Nat Med *25*, 1251-1259. 10.1038/s41591-019-0522-3.
7. Gulley, J.L., Madan, R.A., Pachynski, R., Mulders, P., Sheikh, N.A., Trager, J., and Drake, C.G. (2017). Role of Antigen Spread and Distinctive Characteristics of Immunotherapy in Cancer Treatment. J Natl Cancer Inst *109*. 10.1093/jnci/djw261.
8. Simoni, Y., Becht, E., Fehlings, M., Loh, C.Y., Koo, S.L., Teng, K.W.W., Yeong, J.P.S., Nahar, R., Zhang, T., Kared, H., et al. (2018). Bystander CD8(+) T cells are abundant and phenotypically distinct in human tumour infiltrates. Nature *557*, 575-579. 10.1038/s41586-018-0130-2.

9. Park, S.L., Buzzai, A., Rautela, J., Hor, J.L., Hochheiser, K., Effern, M., McBain, N., Wagner, T., Edwards, J., McConville, R., et al. (2019). Tissue-resident memory CD8(+) T cells promote melanoma-immune equilibrium in skin. Nature *565*, 366-371. 10.1038/s41586-018-0812-9.

10. Gerlinger, M., Rowan, A.J., Horswell, S., Math, M., Larkin, J., Endesfelder, D., Gronroos, E., Martinez, P., Matthews, N., Stewart, A., et al. (2012). Intratumor heterogeneity and branched evolution revealed by multiregion sequencing. N Engl J Med *366*, 883-892. 10.1056/NEJMoa1113205.

11. McGranahan, N., Furness, A.J., Rosenthal, R., Ramskov, S., Lyngaa, R., Saini, S.K., Jamal-Hanjani, M., Wilson, G.A., Birkbak, N.J., Hiley, C.T., et al. (2016). Clonal neoantigens elicit T cell immunoreactivity and sensitivity to immune checkpoint blockade. Science *351*, 1463-1469. 10.1126/science.aaf1490.

12. Kader, T., Lin, J.R., Hug, C.B., Coy, S., Chen, Y.A., de Bruijn, I., Shih, N., Jung, E., Pelletier, R.J., Lopez Leon, M., et al. (2024). Multimodal Spatial Profiling Reveals Immune Suppression and Microenvironment Remodeling in Fallopian Tube Precursors to High-Grade Serous Ovarian Carcinoma. Cancer Discov. 10.1158/2159-8290.CD-24-1366.

13. Stanton, S.E., Castle, P.E., Finn, O.J., Sei, S., and Emens, L.A. (2024). Advances and challenges in cancer immunoprevention and immune interception. J Immunother Cancer *12*. 10.1136/jitc-2023-007815.

RESPONSE TO REVIEWERS' COMMENTS

Reviewer #1 (Remarks to the Author):

Thanks to the authors who have adequately answer to most of the questions and comments.
I only have one last concern about the flow cytometry data. In the scRNAseq analysis, the CD8-SIK3 cluster (Fig 3B) represents a small proportion among TRM cells, which is not confirmed by flow cytometry analysis (Fig S4-5), where a broader proportion of CD8 T cells (or TRM cells?) expressing SIK3 is observed (46% at d0). The authors should comment on this discrepancy?
In the Fig S5 and the flow cytometry analysis, it would be better to see the phenotype changing during stimulation at the T-cell population level, not at each marker individually. What is the exact phenotype switch for the CD8 TRM SIK3 population regarding the exhausted/activation markers compared to the CD8 Tex population? Is the same result observed for TRM and non-TRM populations?

We thank the reviewer for this insightful comment.

Regarding the apparent discrepancy between the scRNA-seq and flow cytometry data, we would like to clarify that the CD8-SIK3 cluster identified in the scRNA-seq analysis represents a transcriptionally defined population characterized by relatively high SIK3 expression compared to other clusters, rather than a strictly binary SIK3-positive versus negative population. In contrast, flow cytometry detects protein expression across a continuous spectrum and classifies cells based on a defined gating threshold. Therefore, while this cluster constitutes a small proportion of TRM cells in the transcriptomic analysis, SIK3 protein expression can still be detected across a broader fraction of CD8 T cells by flow cytometry. This difference in definition likely accounts for the higher proportion (~46% at d0) observed in flow cytometry.

We agree with the reviewer that assessing phenotype changes at the population level would be informative. However, due to the dynamic and gradual nature of SIK3 expression along the inferred pseudotime trajectory (Fig. 3B), it is challenging to define a discrete CD8 TRM SIK3-high population by flow cytometry using a biologically meaningful gating strategy. We therefore analyzed changes in individual activation, proliferation, and exhaustion-associated markers to capture these transitional states.

Finally, regarding the comparison between TRM and non-TRM populations, we acknowledge this as an important point. However, due to the difficulty in reliably defining and isolating discrete SIK3-high TRM populations for downstream analyses, we were unable to determine whether the same phenotypic trends are similarly observed across TRM and non-TRM subsets in this study.

We have clarified these points in the revised manuscript.
