# Supplementary material for: Transcriptomic analysis of tissue-resident memory T cells of the fallopian tube reveals a precursor immune surveillance network for ovarian cancer prevention
Source: Nat Commun. 2026 Jul 13;17:8617. doi: 10.1038/s41467-026-74599-4 (PMC13486501; doi:10.1038/s41467-026-74599-4)
Supplement: Supplementary file 4 [file 41467_2026_74599_MOESM2_ESM.docx]

**Description of Additional Supplementary Files**

**Supplementary Data 1. Differential abundance analysis of TRML clusters across tissues using Speckle/Propeller.** P values were calculated using propeller_ttest or propeller_anova_by_tissue as indicated and adjusted for multiple comparisons using the Benjamini–Hochberg procedure, and adjusted p-values (FDR) < 0.05 were considered statistically significant.

**Supplementary Data 2. Shared clonotype counts and relative abundance of CD8 T-cell subsets across FT, PBMC, and tumor samples.**

**Supplementary Data 3. Shared TCR clonotypes identified across Tem, Tex, and SIK3-high CD8 T-cell subsets.**

**Supplementary Data 4. Differential gene expression analysis of T-cell clusters.** P values were calculated using a two-sided Wilcoxon rank-sum test and adjusted for multiple comparisons using the Bonferroni procedure, and adjusted p-values < 0.05 were considered statistically significant.

**Supplementary Data 5. Gene sets used for gene set correlation analyses, including Shared versus non-shared TCR-associated gene sets, tissue-specific signatures, published NeoTCR8, NeoTCRPBL, and Yost_CD8_Exh gene sets.** Differential expression statistics for the Shared gene set were generated using a two-sided Wilcoxon rank-sum test with Bonferroni-adjusted P values, and adjusted P values < 0.05 were considered statistically significant.
